# Is Smaller Better? Cu^2+^/Cu^+^ Coordination Chemistry and Copper-64 Radiochemical Investigation of a 1,4,7-Triazacyclononane-Based Sulfur-Rich Chelator

**DOI:** 10.1021/acs.inorgchem.3c00621

**Published:** 2023-04-28

**Authors:** Marianna Tosato, Sara Franchi, Abdirisak Ahmed Isse, Alessandro Del Vecchio, Giordano Zanoni, André Alker, Mattia Asti, Thomas Gyr, Valerio Di Marco, Helmut Mäcke

**Affiliations:** † Department of Chemical Sciences, University of Padova, 35131 Padova, Italy; ‡ Roche Pharmaceutical Research and Early Development, Roche Innovation Center Basel F. Hoffmann-La Roche, 4058 Basel, Switzerland; § Radiopharmaceutical Chemistry Section, Nuclear Medicine Unit, AUSL-IRCCS Reggio Emilia, 42122 Reggio Emilia, Italy; ∥ Division of Radiopharmaceutical Chemistry, Clinic of Radiology and Nuclear Medicine, University Hospital Basel, 4031 Basel, Switzerland; ⊥ Department of Nuclear Medicine, University Hospital Freiburg, D-79106 Freiburg, Germany

## Abstract

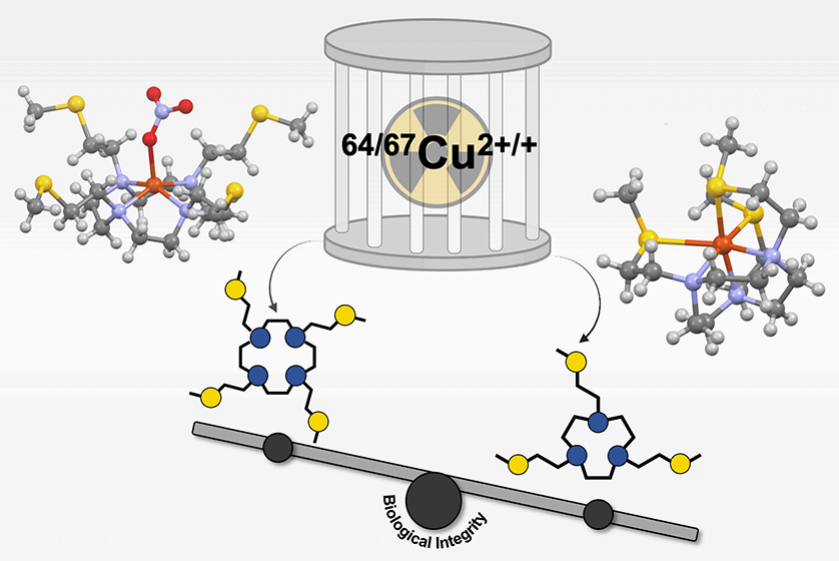

The biologically triggered reduction of Cu^2+^ to Cu^+^ has been postulated as a possible *in vivo* decomplexation pathway in ^64/67^Cu-based radiopharmaceuticals. In an attempt to hinder this phenomenon, we have previously developed a family of S-containing polyazamacrocycles based on 12-, 13-, or 14-membered tetraaza rings able to stabilize both oxidation states. However, despite the high thermodynamic stability of the resulting Cu^2+/+^ complexes, a marked [^64^Cu]Cu^2+^ release was detected in human serum, likely as a result of the partially saturated coordination sphere around the copper center. In the present work, a new hexadentate macrocyclic ligand, 1,4,7-tris[2-(methylsulfanyl)ethyl)]-1,4,7-triazacyclononane (NO3S), was synthesized by hypothesizing that a smaller macrocyclic backbone could thwart the observed demetalation by fully encapsulating the copper ion. To unveil the role of the S donors in the metal binding, the corresponding alkyl analogue 1,4,7-tris-*n*-butyl-1,4,7-triazacyclononane (TACN-*n*-Bu) was considered as comparison. The acid–base properties of the free ligands and the kinetic, thermodynamic, and structural properties of their Cu^2+^ and Cu^+^ complexes were investigated in solution and solid (crystal) states through a combination of spectroscopic and electrochemical techniques. The formation of two stable mononuclear species was detected in aqueous solution for both ligands. The pCu^2+^ value for NO3S at physiological pH was 6 orders of magnitude higher than that computed for TACN-*n*-Bu, pointing out the significant stabilizing contribution arising from the Cu^2+^–S interactions. In both the solid state and solution, Cu^2+^ was fully embedded in the ligand cleft in a hexacoordinated N_3_S_3_ environment. Furthermore, NO3S exhibited a remarkable ability to form a stable complex with Cu^+^ through the involvement of all of the donors in the coordination sphere. Radiolabeling studies evidenced an excellent affinity of NO3S toward [^64^Cu]Cu^2+^, as quantitative incorporation was achieved at high apparent molar activity (∼10 MBq/nmol) and under mild conditions (ambient temperature, neutral pH, 10 min reaction time). Human serum stability assays revealed an increased stability of [^64^Cu][Cu(NO3S)]^2+^ when compared to the corresponding complexes formed by 12-, 13-, or 14-membered tetraaza rings.

## Abstract 2

1,4,7-Tris[2-(methylsulfanyl)ethyl)]-1,4,7-triazacyclononane (NO3S) was synthesized. Its acid−base properties and the kinetic, thermodynamic, and structural properties of its Cu^2+^ and Cu^+^ complexes were investigated in solution and crystal state. NO3S exhibited a remarkable ability to form stable complexes with both Cu^2+^ and Cu^+^ through the involvement of all the donors in the coordination sphere. Radiolabeling studies evidenced an excellent affinity of NO3S towards [6^4^Cu]Cu^2+^ and a remarkable stability of [^64^Cu][Cu(NO3S)]^2+^ in human serum.

## Introduction

When copper radioisotopes, such as copper-61 (^61^Cu, *t*_1/2_ = 3.3 h, *E*_*β*_^+^ = 1.22 MeV, *I*_*β*_^+^ = 61%,*I*_EC_ = 39%), copper-64 (^64^Cu, *t*_1/2_ = 12.7 h, *E*_*β*_^+^ = 655 keV, *I*_*β*_^+^ = 18%;*E*_*β*_^–^ = 573 keV, *I*_*β*_^–^ = 39%) and copper-67 (^67^Cu, *t*_1/2_ = 61.9 h, *E*_*β*_^–^ = 141 keV, *I*_*β*_^–^ = 100%;*E*_γ,1_ = 93 keV, *I*_γ,1_ = 16%;*E*_γ,2_ = 185 keV, *I*_γ,2_ = 49%), are bound to a tumor-targeting moiety through a chelating agent, their emission can be solely directed to the malignant site, providing safe and effective probes for tumor imaging and therapy.^1−7^ A caveat to this paradigm is the capability of the ligand to tightly encapsulate the copper ion to form an extremely thermodynamically stable and kinetically inert complex in biological media. This high stability must be preserved in both copper redox states because Cu^2+^ is likely reduced to Cu^+^ by endogenous reductants.^8−11^ Fast and quantitative radiometal incorporation under mild conditions (i.e., ambient temperature and neutral pH) is also an important outcome when temperature- and/or pH-sensitive biovectors are employed.^12^

To satisfy these requirements, several carboxylate-containing polyazamacrocyclic ligands, such as 1,4,7,10-tetraazacyclododecane-1,4,7,10-tetraacetic acid (DOTA) and 1,4,8,11-tetraazacyclotetradecane-1,4,8,11-tetraacetic acid (TETA) or their rigidified analogues 1,4,7,10-tetraazabicyclo[5.5.2]tetradecane-4,10-diacetic acid (CB-DO2A) and 1,4,8,11-tetraazabicyclo[6.6.2]-hexadecane-4,11-diacetic acid (CB-TE2A), have been developed in the past years (Figure S1). However, the corresponding [^64^Cu]Cu^2+^ complexes exhibited either low *in vivo* stability or necessitated labeling conditions that were too harsh (e.g., *T* > 90 °C), which has precluded their use with thermosensitive biovectors such as antibodies.^2,5,11,13−17^

Several efforts have been devoted to increasing the *in vivo* integrity of the ^64/67^Cu complexes so far. For example, the shrinking of the macrocyclic ring size, resulting in 1,4,7-triazacyclononane-1,4,7-triacetic acid (NOTA), generated one of the current “gold standards” for [^64/67^Cu]Cu^2+^ chelation. NOTA and its functionalized derivative 1,4,7-triazacyclononane-1-glutaric acid-4,7-acetic acid (NODAGA) can bind this radiometal under mild conditions, affording mostly stable complexes *in vivo* (Figure S1).^18^ However, chelating agents suitable for the simultaneous coordination of both copper oxidation states in an endeavor to hamper the radiometal release after the *in vivo* reduction have been rather unexplored so far.^19−21^

**Figure 1:**
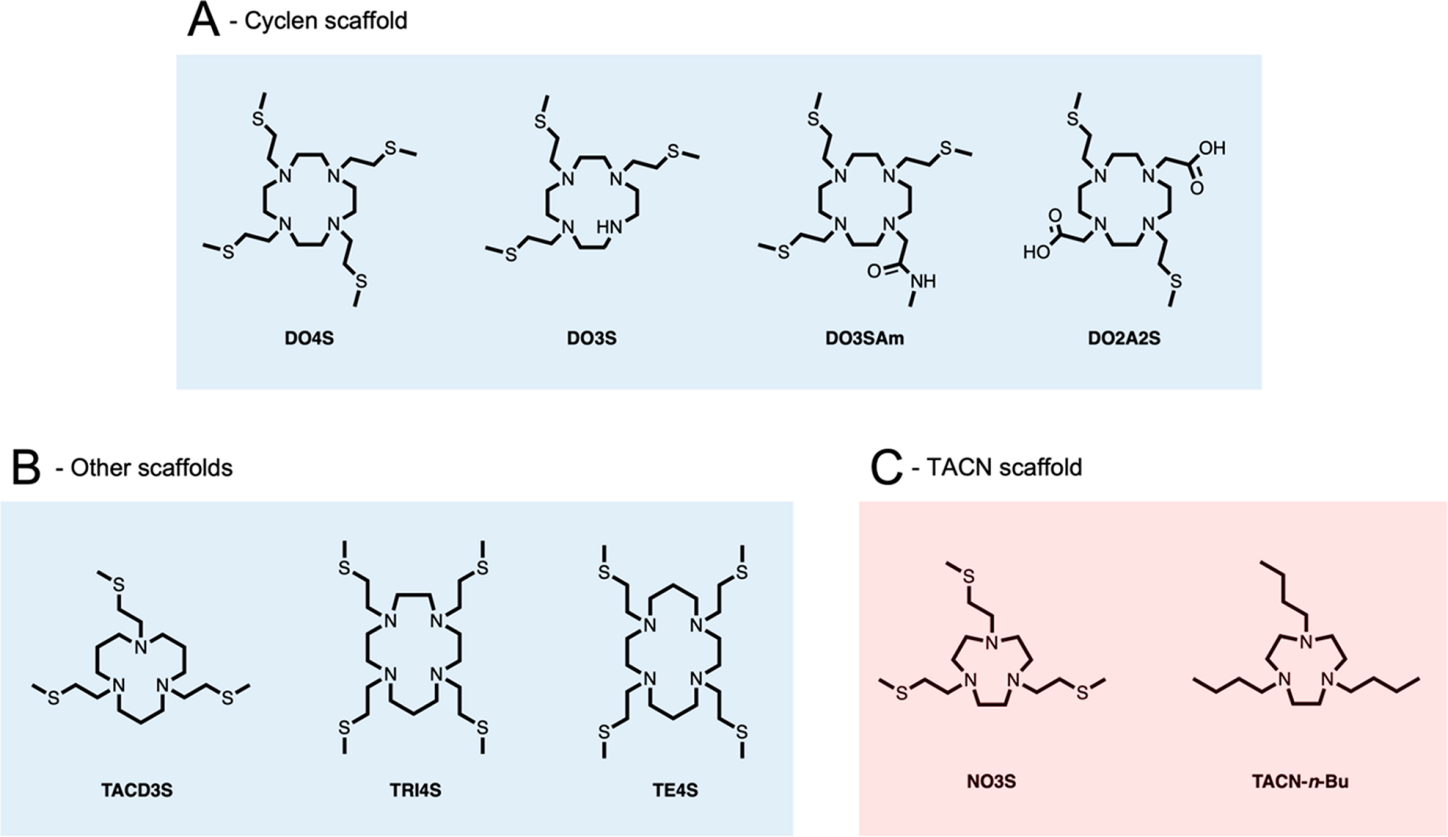
(A) S-containing cyclen-based chelators,^24^ (B) S-containing chelators with variable-ring sizes,^25^ and (C) TACN-based chelators developed and investigated in the present work.

In recent years, with the purpose of pursuing this demand, our research group has pioneered the advance of a new family of sulfanyl-containing macrocyclic ligands for the chelation of borderline and soft, medically interesting radiometals such as [^64/67^Cu]Cu^2+/+^ (Figure 1A,B).^22−29^ The rationale behind the design of these chelators accounted for the fact that the copresence of donor atoms with different chemical softness in the same macrocyclic platform should allow the firm coordination of both the borderline Cu^2+^ and the soft Cu^+^ cations, thus obstructing the undesired demetalation phenomenon.^24−26^

Initially, the 12-membered ring 1,4,7,10-tetraazacyclododecane (cyclen) was fully functionalized with thioether-containing pendant arms, leading to 1,4,7,10-tetrakis-[2-(methylsulfanyl)ethyl]-1,4,7,10-tetraazacyclododecane (DO4S).^24^ DO4S exhibited the ability to stably coordinate both Cu^+^ and Cu^2+^ due to the presence of both N and S donors.^24^ Starting from DO4S, the number of S-containing side chains was progressively decreased or replaced with carboxylic arms to fine tune the whole softness of the generated ligands, resulting in the chelators 1,4,7-tris[2-(methylsulfanyl)ethyl]-1,4,7,10-tetraazacyclododecane (DO3S), 1,4,7-tris[2-(methylsulfanyl)ethyl]-10-acetamido-1,4,7,10-tetraazacyclododecane (DO3SAm), and 1,7-bis[2-(methylsulfanyl)ethyl]-1,4,7,10-tetraazacyclododecane-4,10-diacetic acid (DO2A2S).^24^ While DO3S and DO3SAm formed slightly less stable Cu^2+^/Cu^+^ complexes than DO4S, the stability of the Cu^2+^ complexes produced by DO2A2S was also higher in comparison with that of DOTA, TETA, and NOTA.^24^

Thereafter, we developed another series of macrocycles, namely, 1,5,9-tris[2-(methylsulfanyl)ethyl]-1,5,9-triazacyclododecane (TACD3S), 1,4,7,11-tetrakis-[2-(methylsulfanyl)ethyl]-1,4,7,11-tetraazacyclotridecane (TRI4S), and 1,4,8,11-tetrakis[2-(methylsulfanyl)ethyl]-1,4,8,11-tetraazacyclotetradecane (TE4S), to evaluate the impact of different macrocyclic backbones on the thermodynamic, kinetic, redox, and structural properties of their Cu^2+^/Cu^+^ complexes while maintaining the four 2-(methylsulfanyl)ethyl pendant arms.^25^ Our results revealed that the increase in the macrocyclic ring size (i.e., going from DO4S to TRI4S and TE4S) led to a decrease in both the thermodynamic stability and kinetic inertness, albeit keeping the ability to coordinate both Cu^2+^ and Cu^+^.^25^ The same trend, although quite unexpectedly much more marked, was observed upon increasing the number of carbon atoms between the N donors in TACD3S as well.^25^

Despite the favorable features demonstrated by the pure S-containing ligands developed to date, their [^64^Cu]Cu^2+^ complexes showed rather low integrity in human serum.^26^ We tentatively ascribed this outcome to the partially saturated coordination sphere generated around the metal center that can create open-labile sites.^24,25^ Actually, DFT calculations performed on these ligands indicated that they bind Cu^2+^ with four or a maximum of five donors and that the involvement of additional donors is precluded due to a marked increase in the strain energy.^24^ The resulting vacancies foster the binding of competitive species, thus inducing the observed demetalation.^26^ On these grounds, we considered that S-rich derivatives based on scaffolds equal to or larger than cyclen may not represent the optimal choice for Cu^2+^, and we were propelled to develop a S-containing ligand based on the 1,4,7-triazacyclononane (TACN) backbone, namely, 1,4,7-tris[2-(methylsulfanyl)ethyl)]-1,4,7-triazacyclononane (NO3S, Figure 1C). The good performance displayed by the TACN derivatives (NOTA and NODAGA) toward Cu^2+^ let us hypothesize that the TACN ring could better encapsulate the copper cations affording a fully saturated coordination sphere, thus hindering the possible competitive reactions occurring in biological media. Furthermore, NO3S allows us to complete the evaluation of the macrocyclic backbone effects by exploring a ring smaller than cyclen.

In the present work, the synthesis, acid−base behavior, thermodynamics, kinetics, and structural properties of NO3S toward Cu^2+^ and Cu^+^ are reported. To assess the effect of the sulfanyl pendants on the Cu^2+/+^ coordination, 1,4,7-tris-*n*-butyl-1,4,7-triazacyclononane (TACN-*n*-Bu) was studied for comparison as well (Figure 1C). Collectively, the study was executed through a combination of NMR and UV–vis spectroscopies, X-ray crystallography, and electrochemical techniques. To fully evaluate our hypothesis and appraise the potential of NO3S as a chelator for ^64/67^Cu-based radiopharmaceuticals, its labeling performance and the stability of its [^64^Cu]Cu^2+^ complex in biological media were also investigated.

## Results and Discussion

### NO3S and TACN-*n*-Bu: Synthesis, Acid−Base, and Structural Properties

NO3S was synthesized by complete alkylation of the unsubstituted precursor (i.e., TACN) with 2-chloroethyl methyl sulfide as reported in Figure S2A. The yield is in line with those previously reported for analogous sulfur derivatives.^22−25^ NO3S was fully characterized in nonaqueous solvent by ^1^H and ^13^C{^1^H} nuclear magnetic resonance (NMR) spectroscopy and high-resolution electrospray ionization mass spectrometry (HR-ESI-MS) as detailed in Figures S3–S5. TACN-*n*-Bu was synthesized by the complete alkylation of TACN with 2-bromobutane, as reported in Figure S2B. The full characterization of TACN-*n*-Bu by ^1^H and ^13^C{^1^H} NMR spectroscopy in nonaqueous solvent and HR-ESI-MS is detailed in Figures S6–S8.

Subsequently, the acid–base properties of NO3S and TACN-*n*-Bu were appraised. This study is a prior fundamental step before the investigation of complexation equilibria, since the proton represents a competitor of the metal ion for the interaction with the donor sites having Brønsted and Lewis acid–base properties, such as the amines of the TACN scaffold. The acidity constants (p*K*_a_) of NO3S in aqueous solution were determined at *T* = 25 °C by collecting ^1^H NMR spectra at pH ranging from 0 to 14. The ionic strength (*I*) was adjusted using 0.15 M NaNO_3_. The formation of a precipitate was observed at basic pH because the completely deprotonated neutral form (L, Figure 1C) is sparingly soluble in water. Therefore, additional p*K*_a_ measurements were performed in a 1:1 water/methanol mixture, where no precipitation was detected. Representative ^1^H NMR spectra are reported in Figure S9A, and the signal attributions are summarized in Table S1. The acidity constants of TACN-*n*-Bu were derived upon analysis of the pH-potentiometric titrations data and pH-dependent ^1^H NMR spectra in water (Figure S9B). A detailed description of the proton resonances of TACN-*n*-Bu is reported in Table S2. The acidity constant values of both ligands are gathered in Table 1 along with the values of the unsubstituted macrocycle (i.e., TACN) and the methyl-functionalized one (i.e., 1,4,7-trimethyl-1,4,7-triazacyclonane, TACN-Me) derived from the literature.^30^

**Table 1:** Acidity Constants (p*K*_a_) of NO3S, TACN, TACN-Me, and TACN-*n*-Bu at *T* = 25 °C and *I* = 0.15 M NaNO_3_ (unless otherwise stated)

	p*K*_a_			
Equilibrium reaction^a^	NO3S	TACN^b^	TACN-Me^b^	TACN-*n*-Bu
H_3_L^3+^ ⇌ H^+^ + H_2_L^2+^	-	2.1	–0.4	-
H_2_L^2+^ ⇌ H^+^ + HL^+^	2.70 ± 0.02^c^	6.86	5.1	5.04 ± 0.05^c^
	2.33 ± 0.04^d^			5.0 ± 0.05^e^
HL^+^ ⇌ H^+^ + L	12.72 ± 0.08^c^	10.68	11.7	-f
	11.52 ± 0.01^d^			

a L represents the completely deprotonated form of the ligand as shown in Figure 1C. The reported uncertainty was obtained by the fitting procedure and represents one standard deviation unit.

b From Bianchi et al. (*I* = 0.1 M KNO_3_, *T* = 25 °C).^30^

c Obtained via ^1^H NMR.

d Obtained via pH potentiometry in 1:1 water/methanol.

e Obtained via pH potentiometry.

f Not determined due to precipitation of the sparingly soluble, completely deprotonated L.

In the NMR spectra of NO3S, the SCH_3_ resonance does not undergo significant changes in chemical shift at different pH values, likely because these protons are rather far from the ring, where the sites of protonation/deprotonation are located. However, the chemical shift of all of the other signals manifests a noticeable pH dependence. The most pronounced variation is experienced by the NCH_2_ protons of the ring, possibly because the deprotonation processes led to a marked relaxation of the ring constraint caused by the H^+^−H^+^ repulsions. An analogous pH dependency of the chemical shifts is observed for TACN-*n*-Bu, even if in this case a slowed exchange between the differently protonated forms can be recognized from the enlargement of the signals around pH = p*K*_a,2_. Representative variations of the chemical shift of the different signals as a function of pH are reported in Figures S10 and S11. The data were fitted, and the corresponding protonation constants (Table 1) were obtained. The derived distribution diagrams are shown in Figure S12.

If the p*K*_a_ related to the second deprotonation process occurring in NO3S (p*K*_a,2_ = 2.70) is compared to that of TACN-*n*-Bu (p*K*_a,2_ = 5.0), where each S atom has been replaced by one methylene group, then a noteworthy decrease can be observed (Table 1). Therefore, this behavior must be caused by the S atoms. An analogous effect was also formerly observed with the other S-containing macrocycles displayed in Figure 1A,B, and it was ascribed to the high polarizability and dimension of the S atoms.^22,25^ Actually, it was postulated that sulfurs caused an increased distortion of the ring by hindering the motion of the alkyl chains. This results in an enhanced electrostatic repulsion between the two charged nitrogens of H_2_L^2+^, thus favoring the release of one proton.^22,25^ It is noteworthy that the p*K*_a,2_ of TACN-*n*-Bu is nearly identical to that of TACN-Me, indicating that the length of the alkyl chain has no effect. Moreover, the p*K*_a,2_ values of TACN-*n*-Bu and TACN-Me are lower than that of the unsubstituted macrocycle, (i.e., TACN—p*K*_a,2_ = 6.86, Table 1): this effect is related to the alkylation of the secondary nitrogen atoms of the macrocycle, which destabilizes the generated alkylammonium ion and decreases its water solvation.

**Figure 2:**
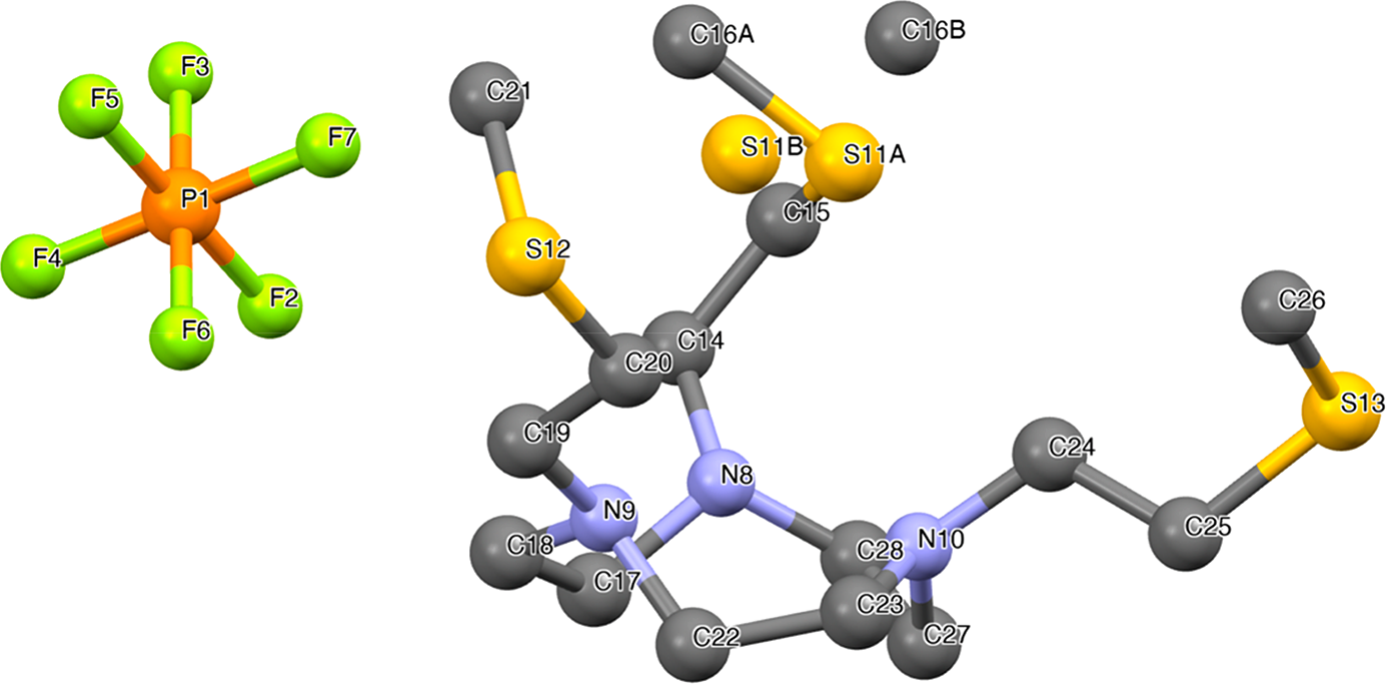
X-ray crystal structure of [H(NO3S)](PF_6_). Atoms marked as S11A-C16A and S11B-C16B represent the possibility of a side chain occupying two different positions.

The solid-state structure of NO3S was determined via X-ray crystallography for crystals of the monoprotonated ligand obtained in CHCl_3_ by the addition of NaPF_6_. Selected bond distances and angles are gathered in Table 2, while crystal data and refinement details as well as further structural information are provided in Tables S3–S5. The solid-state structure of [H(NO3S)](PF_6_) is shown in Figure 2. As can be noticed, all of the S-containing chains are *syn* oriented, but the thioether arm on the opposite side with respect to the counterion slightly expands the cleft formed by the N_3_S_3_ set of donors with an asymmetrical position. This is likely due to the small TACN ring size that hinders a closer contact among the arms. In fact, this distorted arrangement was not detected in the 12-membered-ring analogue DO4S and can be rationalized from a steric point of view since the neighboring N atoms are 2.918 and 2.782 Å apart in DO4S and NO3S, respectively.^31^ As a result, in NO3S, the free-to-move side chains expand to circumvent this steric constraint. Intriguingly, two slightly different conformations have been refined for one of the S-containing side chains of NO3S (Figure 2).

**Table 2:** Selected Representative Bond Lengths and Angles of [H(NO3S)](PF_6_) and [Cu(NO3S)][Cu(NO_3_)_4_]^a^

[H(NO3S)](PF_6_)			
Bond	Bond length [Å]	Angle	Degrees
N(8)–C(28)	1.485(9)	C(28)–N(8)–C(17)	111.8(5)
N(8)–C(17)	1.507(9)	C(28)–N(8)–C(14)	114.4(5)
N(8)–C(14)	1.514(10)	C(17)–N(8)–C(14)	112.5(6)
N(9)–C(22)	1.471(8)	C(15)–S(11A)–C(16A)	96.6(7)
N(10)–C(23)	1.467(9)	C(16B)–S(11B)–C(15)	97.0(9)
S(11A)–C(15)	1.72(10)	C(20)–S(12)–C(21)	100.4(3)
S(11A)–C(16A)	1.82(2)	C(14)–C(15)–S(11A)	122.6(7)
S(11B)–C(16B)	1.82(2)	C(14)–C(15)–S(11B)	102.6(7)
S(11B)–C(15)	1.957(12)	C(19)–C(20)–S(12)	112.4(4)
S(12)–C(20)	1.799(6)	C(24)–C(25)–S(13)	112.6(5)
S(12)–C(21)	1.804(7)		

[Cu(NO3S)][Cu(NO_3_)_4_]			
Bond	Bond length [Å]	Angle	Degrees
Cu(1)–N(3)	2.064(12)	N(3)–Cu(1)–N(1)	84.1(5)
Cu(1)–N(2)	2.153(12)	N(3)–Cu(1)–S(1)	169.6(3)
Cu(1)–N(1)	2.186(11)	N(3)–Cu(1)–S(2)	85.0(3)
Cu(1)–S(1)	2.383(4)	N(3)–Cu(1)–S(3)	96.8(3)
Cu(1)–S(2)	2.476(4)	S(1)–Cu(1)–S(3)	93.2(2)
Cu(1)–S(3)	2.727(5)	S(1)–Cu(1)–S(2)	96.69(14)
S(1)–C(7)	1.82(2)	S(1)–Cu(1)–S(3)	93.2(2)
S(2)–C(13)	1.80(2)	C(8)–S(1)–C(7)	101.3(8)
S(3)–C(3)	1.81(2)	C(8)–S(1)–Cu(1)	110.6(6)
Cu(2)–O(101)	1.950(11)	C(7)–S(1)–Cu(1)	98.8(5)
Cu(2)–O(104)	1.959(11)	C(7)–S(1)–Cu(1)	98.8(5)

a See Figures 2 and 6 for atom labeling.

### Cu^2+^-NO3S and Cu^2+^-TACN-*n*-Bu: Complexation Kinetics

The formation kinetics of the Cu^2+^ complexes with NO3S and TACN-*n*-Bu was qualitatively appraised by UV–vis spectroscopy at ambient temperature and different pH values to explore the time necessary to reach equilibrium. Representative variations of the absorbance over time and the time courses of the complexation reactions are shown in Figures S13 and S14. The spectrum of NO3S and TACN-*n*-Bu as free ligands is also reported for comparison purposes in Figure S15.

The acquired data revealed that, at equimolar metal-to-ligand concentrations (10^–4^ M), NO3S can quickly bind Cu^2+^ at pH ≥ 4 as the equilibrium occurred in a few minutes (e.g., ∼10 min at pH = 4.0 and <1 min at pH = 7.1) while, at more acidic pH, the complexation kinetics were slower (e.g., ∼14 h at pH = 1.0). This slowed-down reactivity can be justified by considering the progressively more intense electrostatic repulsions generated between the N-bound H^+^ of the differently protonated ligand forms and the incoming Cu^2+^ cation. The charged nitrogens hinder the entry of the metal into the binding cleft, as also previously observed with the other S-rich polyazamacrocycles in Figure 1A,B.^24,25^ If the complexation kinetics of all of the S-rich chelators reported so far are directly compared, then NO3S exhibits the best performance at all pH values.

Furthermore, the kinetic investigation revealed that the time necessary to reach equilibrium was appreciably longer when the S-side chains of NO3S were replaced by the *n*-butyl substituents in TACN-*n*-Bu: for example, the complexation reaction with TACN-*n*-Bu at neutral pH occurred in 8 h (vs <1 min with NO3S), as shown in Figure S14. This result points out the existence of a bonding interaction between the S donors and the metal cation. It can be hypothesized that the preorganization of NO3S, as detected in the solid state (*vide supra*), allows some or all S donors to efficiently interact with Cu^2+^ forming an out-of-sphere complex. Afterward, this complex is evolved in the final geometry, thereby increasing the local Cu^2+^ concentration around the N donor and accelerating the complexation event compared to TACN-*n*-Bu.

### Cu^2+^-NO3S and Cu^2+^-TACN-*n*-Bu: Complexation Thermodynamics

The stability constants (log*β*) of the Cu^2+^ complexes formed by NO3S and TACN-*n*-Bu were determined via UV–vis spectrophotometric titrations. Due to both the slow complexation kinetics observed at acidic pH (*vide supra*) and the high complex stability, batch titrations were conducted and heating was applied to speed up reaching the equilibrium condition.

**Figure 3:**
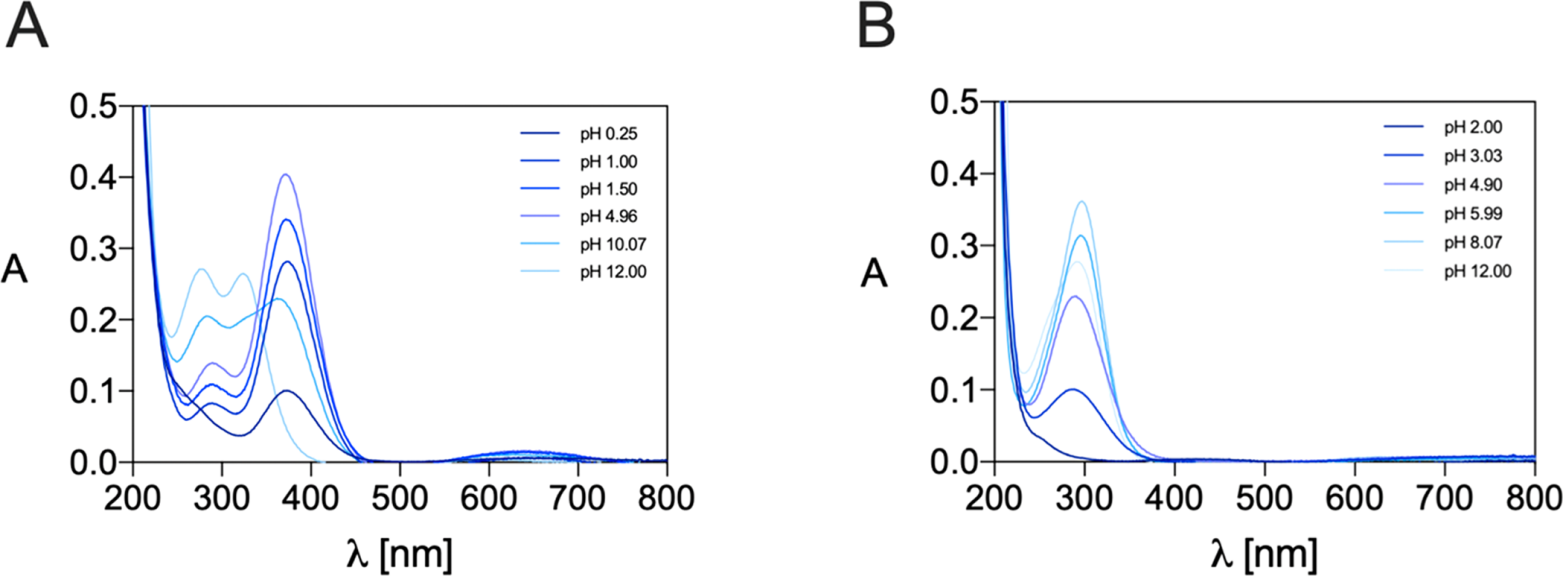
Representative UV–vis spectra of the Cu^2+^ complexes formed by (A) NO3S and (B) TACN-*n*-Bu at different pH values (*C*_L_ = *C*_Cu_^2+^ = 1.0 × 10^–4^ M, *I* = 0.15 M NaNO_3_ at pH < 1, the ionic strength was not controlled, and *T* = 25 °C).

As shown in Figure 3A, after the addition of Cu^2+^ to NO3S, from acidic to neutral pH, a distinct change in the electronic spectra with respect to the free ligand (reported in Figure S15A) was recognizable.

The change was evidenced by the appearance of three electronic transitions with a maximum absorption at λ_max_ = 372 nm, indicative of the complexation event. These absorbances experienced a shift toward higher energies at more basic pH (Figure 3A). Furthermore, an isosbestic point is recognizable at λ = 340 nm (except at very acidic pH), highlighting the existence of two different cupric complexes, one predominant at acidic-neutral pH and the other under basic pH conditions. The variation of the absorbance at 372 nm as a function of pH is shown in Figure S16A.

**Figure 4:**
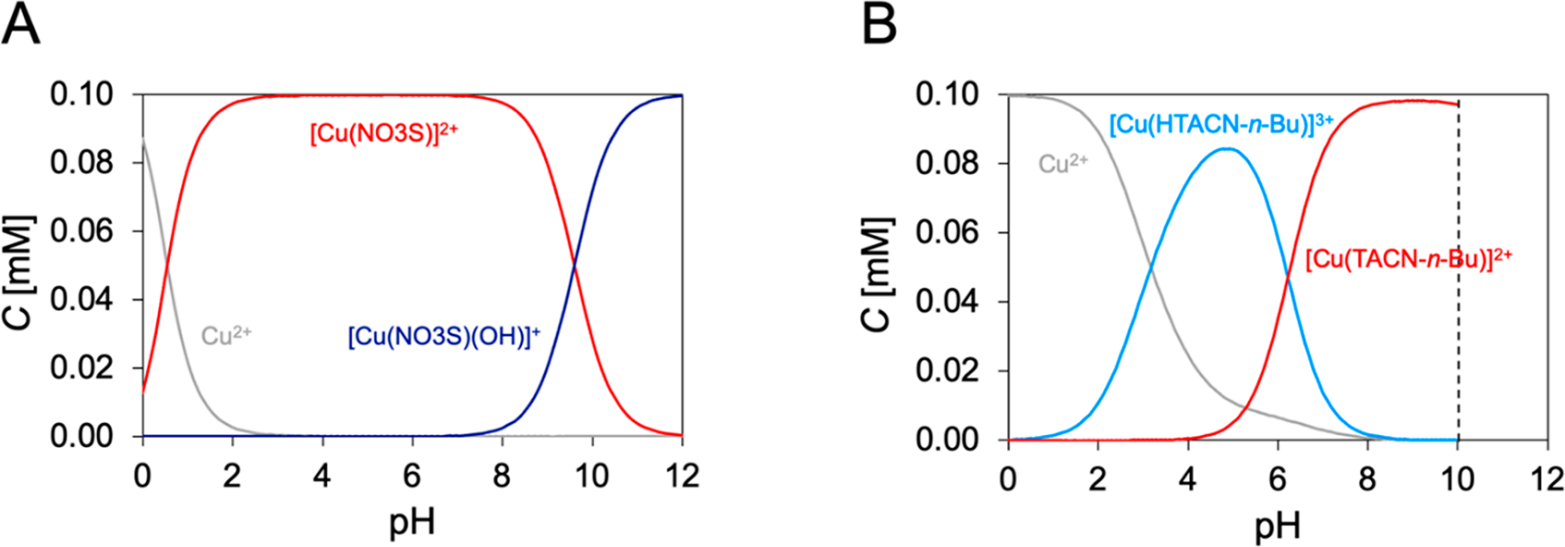
Speciation diagram of Cu^2+^ and (A) NO3S and (B) TACN-*n*-Bu (*C*_L_ = *C*_Cu_^2+^ = 1 × 10^–4^ M). The dashed line represents the formation of the slightly soluble species.

The stoichiometry of these two complexes was determined via UV−vis spectrophotometric titrations at different Cu^2+^-to-NO3S molar ratios and pH and via HR-ESI-MS analysis, as reported in Figure S17–S19. The metal-to-ligand ratio resulted 1:1 and the subsequent data treatment indicated their speciation as [CuL]^2+^ and [CuL(OH)]^+^, respectively. The overall formation constants are reported in Table 3 while an example of speciation diagram is shown in Figure 4A.

**Table 3:** Overall Stability Constants (log β) of the Cu^2+^ and Cu^+^ Complexes Formed by NO3S and TACN-*n*-Bu at *T* = 25 °C and *I* = 0.15 M NaNO_3_^a^

	log*β*	
Equilibrium reaction^a^	NO3S	TACN-*n*-Bu
Cu^2+^ + HL^+^ ⇌ [CuHL]^3+^^b^	-	6.2 ± 0.1
Cu^2+^ + HL^+^ ⇌ [CuL]^2+^ + H^+^^b^	-	–0.04 ± 0.15
Cu^2+^ + L ⇌ [CuL]^2+^^b^	18.41 ± 0.05	-
Cu^2+^ + L + H_2_O ⇌ [CuL(OH)]^+^ + H^+^^b^	9.08 ± 0.03	-
Cu^+^ + L ⇌ [CuL]^+^^c^	18.36 ± 0.05	-

a L represents the completely deprotonated form of the ligand as shown in Figure 1C. The reported uncertainty was obtained by the fitting procedure and represents one standard deviation unit.

b Obtained by UV–vis spectrophotometric titrations.

c Obtained from voltametric data.

**Figure 5:**
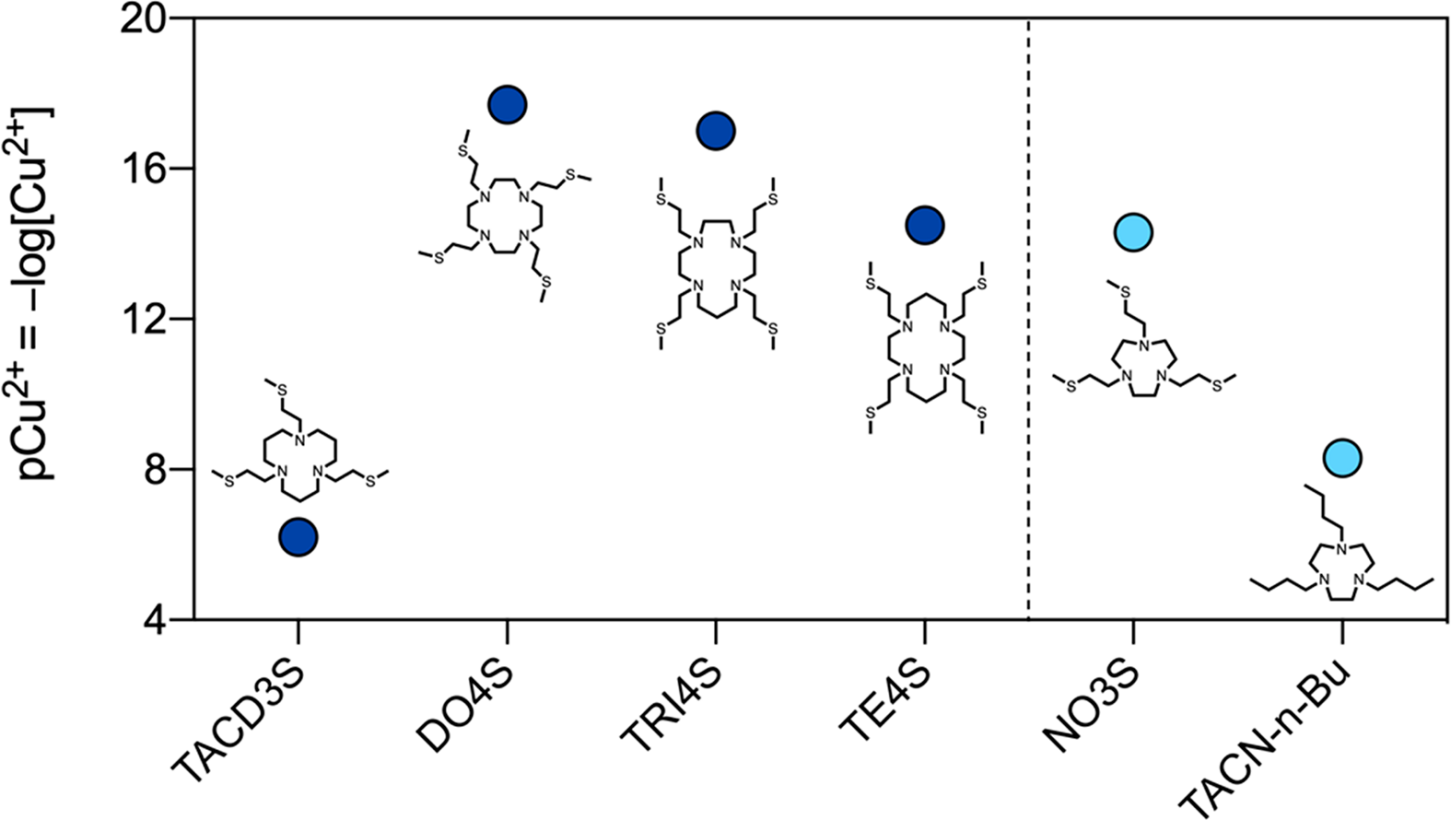
Comparison of the pCu^2+^ values for the S-containing ligands investigated in previous works (DO4S, TRI4S, TE4S, and TACD3S) with NO3S and TACN-*n*-Bu. The reported pCu^2+^ values were calculated at *C*_L_ = 1 × 10^–5^ M and *C*_Cu_^2+^ = 1 × 10^–6^ M and pH 7.4 using the stability constants reported in Tables 1 and 3 or taken from the literature.^24,25^

The stability of the Cu^2+^ complexes with TACN-*n*-Bu was assessed as well to investigate the role of the sulfanyl pendants in metal coordination. The UV–vis spectra recorded at different pH values are shown in Figure 3B, and the variation of the absorbance at λ = 295 nm is reported in Figure S16B. The results obtained are shown in Table 3 and Figure 4B. The pCu^2+^ values were then computed (Figure 5): being defined as −log[Cu^2+^]_free_, the higher the pCu^2+^ value, the greater the stability of the considered metal–ligand complex.^23−25^ As shown in Figure 4B and Figure 5, the presence of S in NO3S afforded not only a significant speciation change but also an extraordinary increase in the complex stability (6 orders of magnitude) with respect to TACN-*n*-Bu, thus demonstrating the key role of the sulfur donors in Cu^2+^ stabilization.

The pCu^2+^ calculation also allowed us to assess the effect of decreasing the ring size and number of N donors on the complex stability. When the performances of all of the pure S-containing analogues are compared, the stability ranks in the following order: DO4S ≥ TRI4S > TE4S ≅ NO3S ≫ TACD3S (Figure 5). Although NO3S is not the chelator with the highest stability of the series, its pCu^2+^ of 14.3 is still high enough to warrant further radiochemical investigations. The Cu^2+^-NO3S complexes are less stable also if compared with the Cu^2+^-NOTA ones (NOTA has the same ring scaffold as NO3S, but sulfanyl arms are replaced by carboxylates; see Figure S1). As for NOTA, a pCu^2+^ value of 18.2 can be computed at pH = 7.4. This indicates a preference of Cu^2+^ toward carboxylates rather than toward sulfur. However, the decreased stability of the Cu^2+^ complexes formed by NO3S, when compared to that of NOTA, should be balanced by an increased stability of its Cu^+^ complexes. Moreover, even if high thermodynamic stability is one of the key properties of a metal complex for *in vivo* applications, kinetic inertness might become the leading factor that guides its integrity in biological environments (*vide infra*).

### Cu^2+^-NO3S: Solid-State and Solution Structures

**Figure 6:**
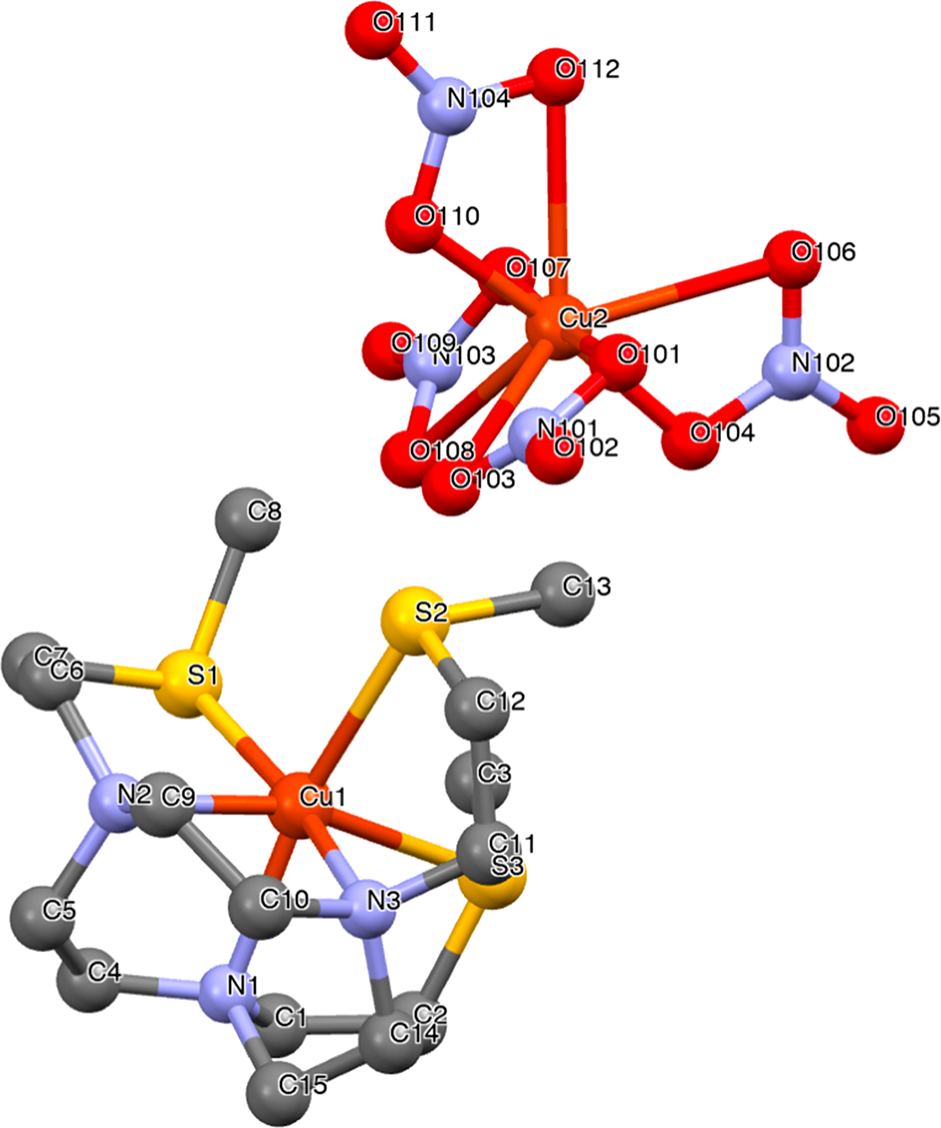
X-ray crystal structure of [Cu(NO3S)][Cu(NO_3_)_4_].

The structure of Cu^2+^-NO3S was explored in the solid state by single-crystal X-ray diffraction. A view of the crystal structure of [Cu(NO3S)][Cu(NO_3_)_4_] is shown in Figure 6, and selected representative bond distances and angles are gathered in Table 2. Crystal data and refinement details as well as further structural information are provided in Tables S6 and S8.

In [Cu(NO3S)]^2+^, Cu^2+^ is deeply embedded in the cleft formed by the three N donors of the macrocyclic ring and the three S atoms of the pendant chains in a symmetric N_3_S_3_ hexacoordinate environment. The copper ion is 1.36 Å above the N3 plane and 1.31 Å below the S3 plane, and the S3 plane is rotated 46.9° clockwise with respect to the N3 plane. The average bond lengths are equal to 2.13 ± 0.03 Å for Cu–N and 2.53 ± 0.06 Å for Cu–S. On the other hand, the autonomous [Cu(NO_3_)_4_]^2–^ complex is located 8.3 Å (Cu^1^–Cu^2^ distance) from [Cu(NO3S)]^2+^, and Cu^2+^ is coordinated by the four nitrate anions. However, in Cu^2+^-DO4S, each Cu^2+^ was surrounded by four nitrogens of the macrocyclic ring (average N–Cu^2+^ bond distances equal to 2.04 Å) and a nitrate anion in square-pyramidal geometry, while S did not form any bond with the metal center.^24^

In aqueous solution, the electronic spectrum of [Cu(NO3S)]^2+^ displayed a very intense band centered at λ_max_ = 372 nm (ε_372 nm_ = 4.0 × 10^3^ mol^–1^·cm^–1^·L calculated from Lambert–Beer’s law, ε_372 nm_ = (3.5 ± 0.1) × 10^3^ mol^–1^· cm^–1^·L computed from data treatment) accompanied by two less-intense UV and visible absorptions at λ = 285 nm (ε_285 nm_ = 1.4 × 10^3^ mol^–1^· cm^–1^·L from Lambert–Beer’s law) and λ = 635 nm (ε_635 nm_ = 1.6 × 10^2^ mol^–1^·cm^–1^·L from Lambert–Beer’s law). While the latter is characteristic of the *d*–*d* orbital transition of the metal center, the band at λ = 372 nm can be attributed to a S-to-Cu^2+^ ligand-to-metal charge-transfer transition (based on the literature^24^), thus further pointing out the existence of the S–Cu interaction in solution. Intriguingly, this absorption is shifted toward lower energy with respect to [Cu(DO4S)]^2+^ or [Cu(TE4S)]^2+^ which in solution possesses a mixed N_4_ + N_4_S_ax_ and a pure N_4_S_ax_ coordination sphere, respectively (Figure S20).^24^ This confirms that NO3S has a structural arrangement around Cu^2+^ that differs from that of the other two ligands.

Regarding the adsorption at λ = 285 nm, as shown in Figure S21, the same UV transition can be recognized if the spectra of [Cu(NO3S)]^2+^ and its alkyl analogue [Cu(TACN-*n*-Bu)]^2+^ are compared. Therefore, this band can likely be attributed to a N-to-Cu^2+^ CT transition since TACN-*n*-Bu can provide only N donors. This finding further supports the attribution of the band at λ = 372 nm of [Cu(NO3S)]^2+^ to a S-to-Cu^2+^ transition; consequently, the [Cu(NO3S)]^2+^ electronic spectrum appears as the convolution of the S-to-Cu^2+^ and N-to-Cu^2+^ transitions. In conclusion, it can be postulated that the hexacoordinate structure detected in the solid state (*vide supra*), in which the metal ion is encapsulated in N_3_S_3_ geometry, is maintained in aqueous solution.

### Cu^+^-NO3S: Evaluation of the Short- and Long-Term Stability

#### Short-Term Stability

**Figure 7:**
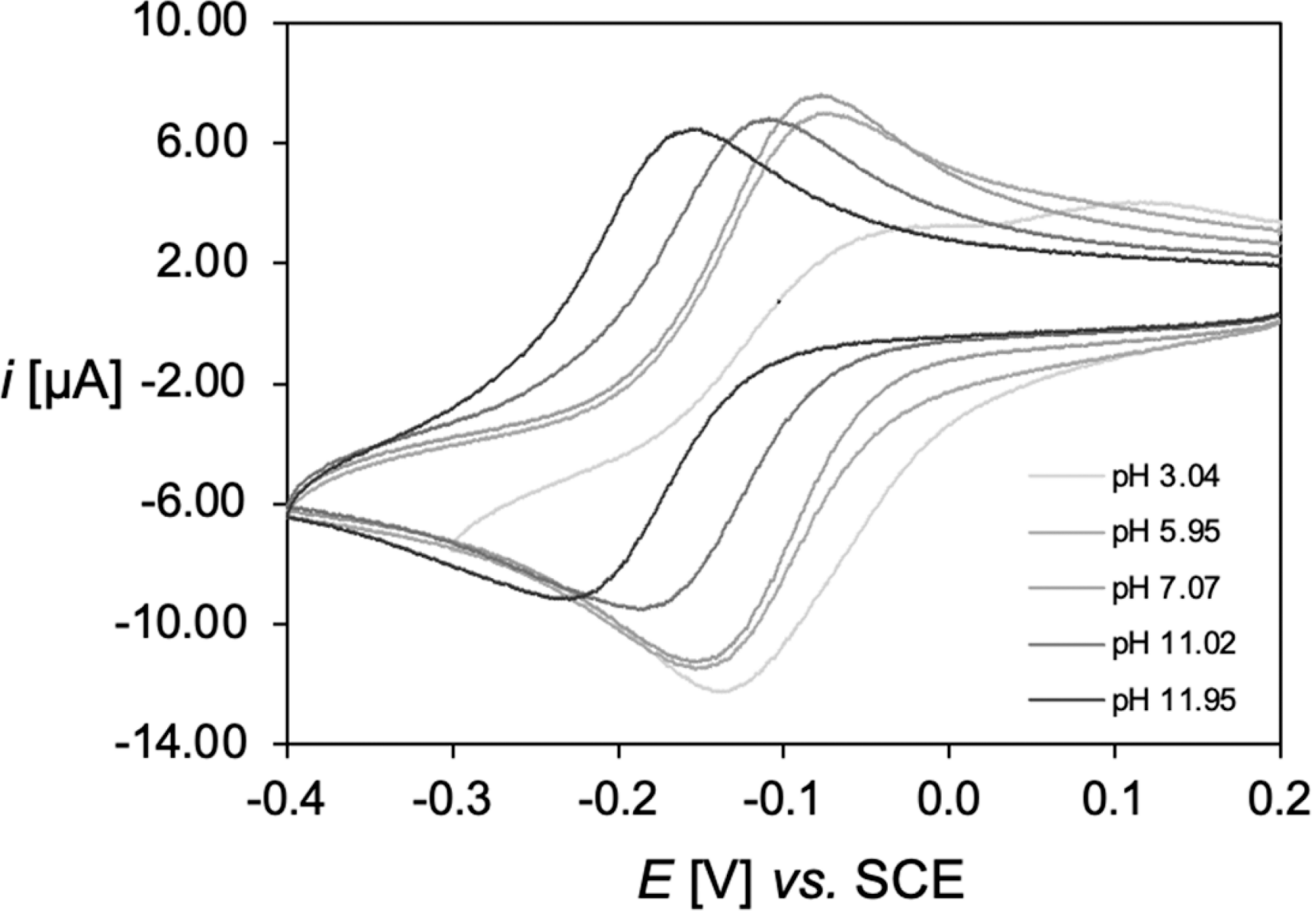
Representative cyclic voltammograms of the copper complex of NO3S (*C*_[Cu(NO3S)]_^2+^ = 9.8 × 10^–4^ M) in aqueous solution at different pH values, with *I* = 0.15 M NaNO_3_ and *T* = 25 °C, acquired at a scan rate (*v*) of 0.1 V/s.

To assess the ability of NO3S to stably bind Cu^+^, cyclic voltammetry (CV) analysis was executed. The investigation was performed in aqueous solution at pH ranging from 3 to 12. The obtained cyclic voltammograms, reported in Figure 7, show a peak couple attributable to the quasi-reversible one-electron reduction–oxidation process of the Cu^2+^/Cu^+^ redox pair (Table S9). No variation of the voltammetric behavior after multiple reduction–oxidation cycles was observed either with time or with the scan rate (except for the increase in the current intensity, *i*). This outcome is extremely significant because it proves the ability of NO3S to stabilize both copper oxidation states throughout the investigated pH range on the time scale of the CV experiments.

As illustrated in Figure 7 and Table S9, a pH-dependent variation of the cyclic voltammograms was found. At 4 ≤ pH ≤ 9, the cathodic (*E*_p,c_) and anodic peak potentials (*E*_p,a_) are independent of the proton content of the solution (Figure S22), thus implying that a single Cu^2+^/Cu^+^ pair exists in this pH range. The Cu^2+^ species is [Cu(NO3S)]^2+^ (*vide supra*), and we assumed that the Cu^+^ species is [Cu(NO3S)]^+^ as previously observed with the other S-containing polyazamacrocycles.^24,25^ The standard potential of the [Cu(NO3S)]^2+^/[Cu(NO3S)]^+^ couple was estimated from the peak potentials as *E*_1/2_ = (*E*_pa_ + *E*_pc_)/2. At pH > 9, changes in the voltammetric pattern begin to be observable as the peak potentials shift toward more negative values (Figure S22). This behavior can be explained by taking into consideration the Cu^2+^ speciation, according to which the formation of [Cu(NO3S)(OH)]^+^ starts to occur at basic pH (*vide supra*).

At pH < 4, some variations are also noticeable (Figure 7 and Figure S22). However, pH values lower than 3 were not examined, as free Cu^2+^ starts to form. Remarkably, the pH variations do not affect the kinetics of the electron transfer (ET) which was always quite fast, with Δ*E*_p_ = *E*_pa_ – *E*_pc_ values slightly higher than the canonical 60 mV for Nernstian ET processes, as reported in Table S9.

**Figure 8:**
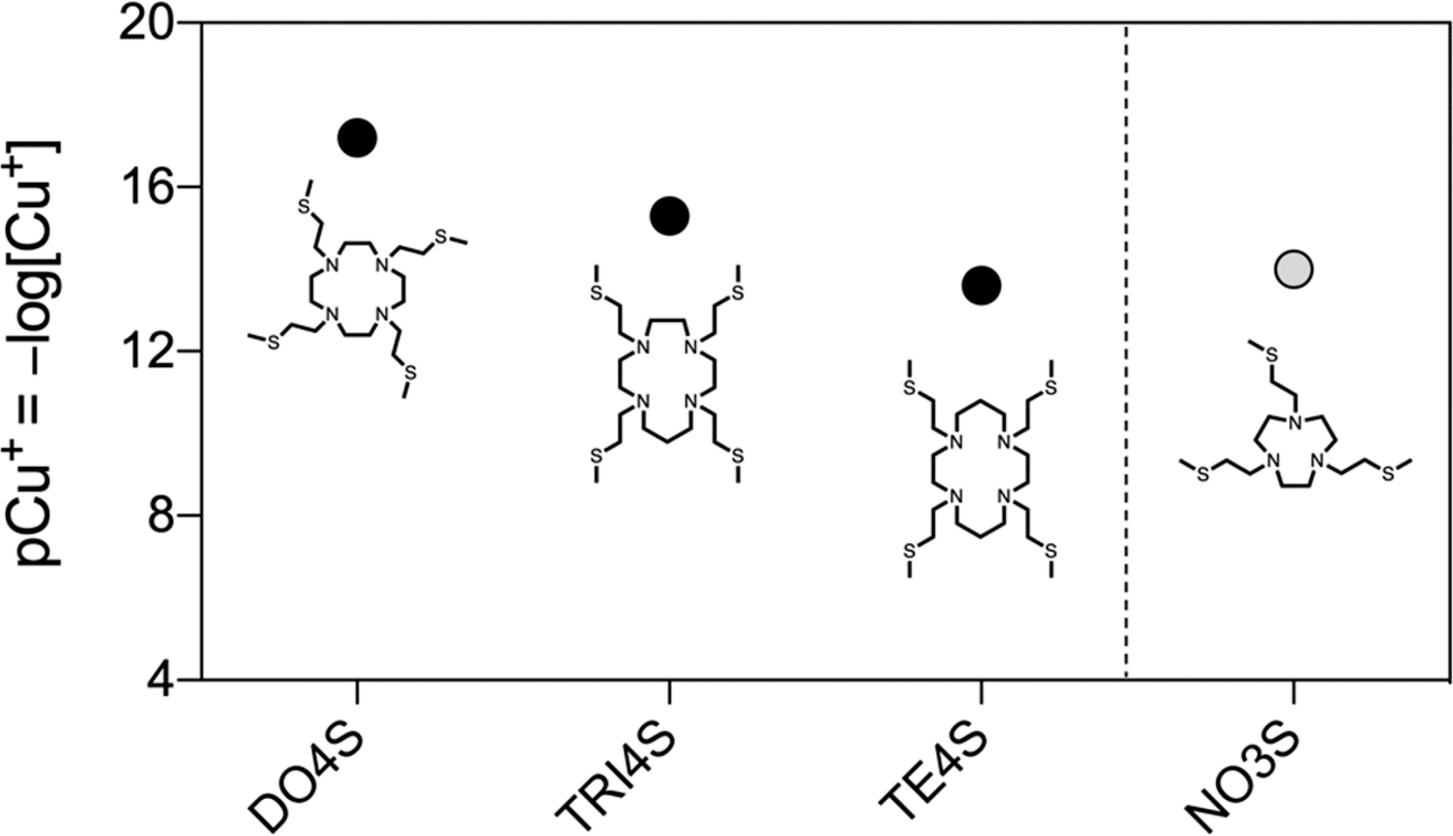
Comparison of the pCu^+^ values for the S-containing ligands investigated in the previous works (DO4S, TRI4S, and TE4S) with NO3S. The reported pCu^+^ values were calculated at *C*_L_ = 10^–5^ M and *C*_Cu_^+^ = 10^–6^ M at pH = 7.4 using the stability constants reported in Table 3 or taken from the literature.^24,25^

The stability constant of [Cu(NO3S)]^+^ obtained from the cyclic voltammetric data is reported in Table 3. The computed pCu^+^ is very large (14.0), and it is comparable to that obtained for Cu^2+^. If compared to the previously developed S-containing chelators, the pCu^+^ also followed a trend similar to that already reported for Cu^2+^ with DO4S > TRI4S > NO3S > TE4S (Figure 8). No stability is reported in the literature for the Cu^+^ complexes formed by NOTA, but it is expected that they are much less stable than those formed by NO3S due to the well-known preference of Cu^+^ for soft donors such as sulfur rather than for hard donors such as carboxylates.

As a final consideration, by comparing the standard reduction potential (*E*^0^) of [Cu(NO3S)]^2+^ at physiological pH (assuming *E*^0^_Cu/NO3S_ = *E*_1/2,Cu/NO3S_ = −0.121 V vs SCE) with the threshold *E*^0^ of the common biological reducing agents (*E*^0^ = −0.64 V vs SCE), it is readily apparent that it is prone to *in vivo* reduction.^2^ However, as demonstrated by CV and bulk electrolysis experiments (*vide infra*), the cleavage of the complex should be prevented by the capability of NO3S to stabilize both Cu^2+^ and Cu^+^.

#### Long-Term Stability

To judge the long-term stability of [Cu(NO3S)]^+^ and gain further insight into its solution speciation, bulk electrolysis of Cu^2+^-NO3S solutions was performed at several pH values, followed by the collection of ^1^H NMR spectra. The marked spectral variation when compared with that of free NO3S (Figure S23) confirms the ability of the chelator to bind Cu^+^ even during long time intervals. Additionally, the absence of pH-dependent spectral variations (Figure S24) points to the existence of a unique Cu^+^ complex in the investigated pH range. The protons on the S-bound carbons (SCH_3_ and SCH_2_) are equivalent on the NMR time scale since they resonate as a singlet at 2.22 ppm and an asymmetric triplet at 2.87 ppm and experience a downfield shift upon metal binding (Table S10). The latter spectral feature proves the existence of Cu^+^–S bonds in solution, and their fine structure suggests that all of the S atoms are either statically bound to the metal center or are in rapid exchange during the time scale of the NMR experiment. The N-bound CH_2_ of the pendant arms resonates as a single asymmetric triplet at 2.94 ppm, shielded with respect to the free ligand, implying either the concomitant static role of all of the N atoms in the metal binding or their rapid solution exchange. The NCH_2_ protons in the macrocyclic backbone are shielded with respect to the free ligand and are split into two quasi-symmetric multiplets centered at 2.65 and 2.80 ppm (Figure S23), indicating that the Cu^+^ binding induces the magnetic nonequivalence of the ethylic fragments (which may be attributed to the axial and equatorial orientations of ring protons). The higher electron density experienced by the N-bound CH_2_ protons of [Cu(NO3S)]^+^ as compared to NO3S could be justified by considering that, while in the free ligand H^+^ is located solely on the N atoms, in the complex the Cu^+^ ion is concurrently interacting with both N and S donors, thus sharing the +1 charge among all of them.

Combining all of these spectral features with the quasi-reversibility of the cyclic voltammograms, which indicates that the coordination environment around copper is rather unchanged upon the variation of the oxidation state, an average N_3_S_3_ coordination environment can be assumed for [Cu(NO3S)]^+^.

### [^64^Cu]Cu^2+^-NO3S: Radiolabeling and Human Serum Integrity

To assess the ability of NO3S to chelate [^64^Cu]Cu^2+^ under extremely dilute radiochemical conditions, a radiolabeling investigation was conducted to explore the effects of temperature, pH, and chelator concentration on the radiochemical incorporation (RCI). Parallel experiments were performed as well using NODAGA, one of the current gold standards for [^64^Cu]Cu^2+^ chelation endowing a NOTA moiety, for comparison purposes. This ligand was also chosen as it contains the same TACN backbone of NO3S. The RCI was determined using both radio-UHPLC and radio-TLC analysis; a representative HPLC radiochromatogram is reported in Figure S25.

**Figure 9:**
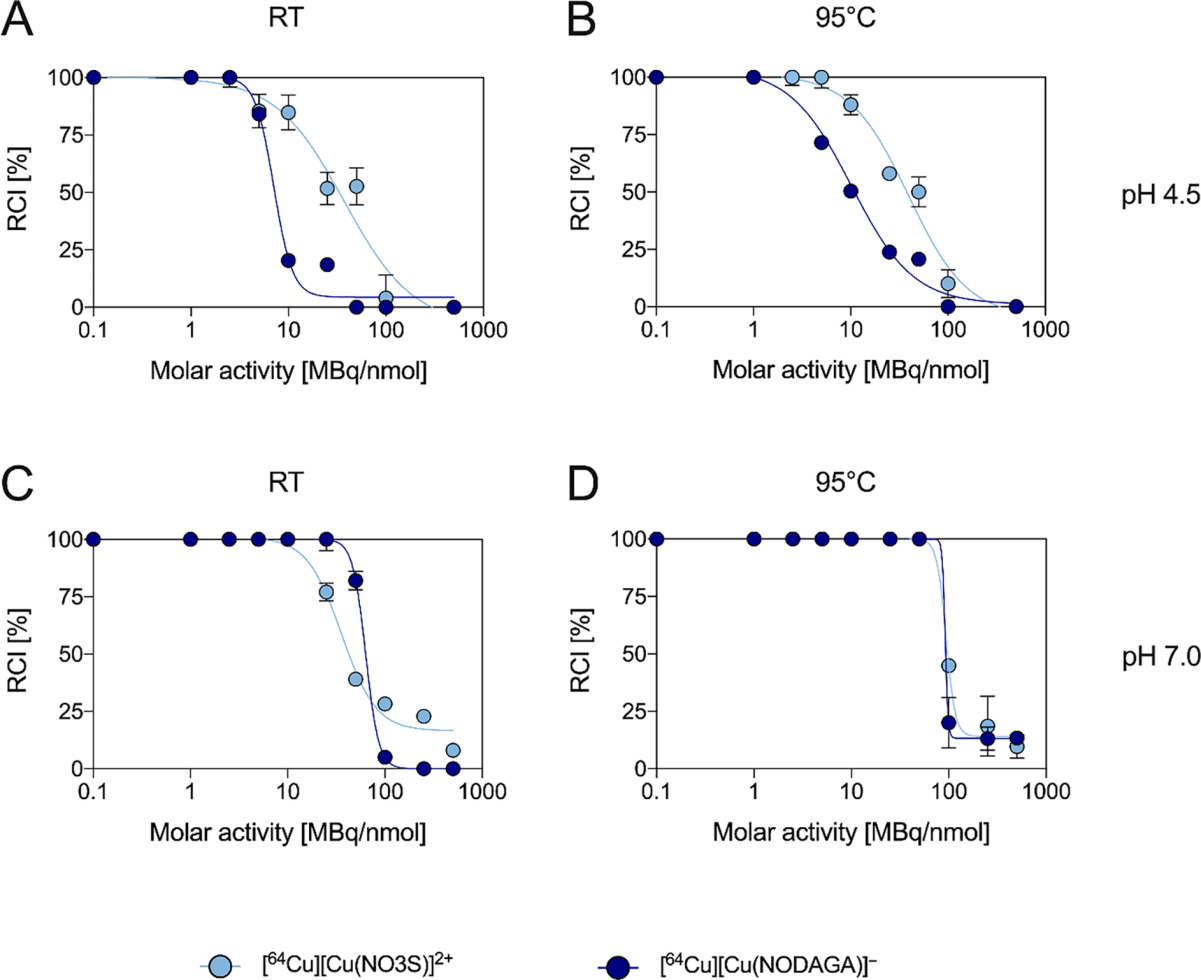
Comparison of the [^64^Cu]Cu^2+^ incorporation yields at different molar activities for NO3S and NODAGA at pH = 4.5 and (A) RT and (B) 95 °C and at pH = 7.0 and (C) RT and (D) 95 °C (10 min reaction time).

As displayed in Figure 9A, at room temperature and acidic pH (pH = 4.5) NO3S was able to quantitatively incorporate [^64^Cu]Cu^2+^ (RCI > 99%) at an apparent molar activity of up to 2.5 MBq/nmol in 10 min. The RCI progressively decreased when increasing the molar activity (i.e., lowering the chelator concentration) and dropped to <5% at molar activities >100 MBq/nmol. Although NODAGA achieved quantitative labeling at nearly the same maximum molar activity as did NO3S (i.e., 2.5 MBq/nmol), it demonstrated a lower labeling efficiency (Figure 9A). If compared with DO4S (i.e., our previously best-performing full S-substituted polyazamacrocycle), then NO3S showed superior features of being able to achieve quantitative incorporation at molar activity more than 2-fold higher.^26^

When the temperature was increased to 95 °C (Figure 9B), a slight enhancement of the performances of NO3S was observed (e.g., the quantitative labeling shifted from 2.5 MBq/nmol to around 5 MBq/nmol), and for NODAGA, the effect of the temperature variation on the maximal molar activity was less impacting. However, an improvement in its labeling trend was observed (e.g., the RCI shifted from 25% at RT to 50% at 95 °C at 10 MBq/nmol) (Figure 9A,B). As a result, NO3S still exceeded the performance of NODAGA at this temperature as well.

The change in pH from a mildly acidic to a neutral environment strongly enhanced the [^64^Cu]Cu^2+^ incorporation of NODAGA. Under this condition and at ambient temperature (Figure 9C), NODAGA was able to quantitatively incorporate the radiometal at a 10-fold-higher apparent molar activity than at pH = 4.5 (i.e., 25 MBq/nmol), slightly surpassing the performance of NO3S (quantitative incorporation around 10 MBq/nmol). At 50 MBq/nmol, the RCI with NODAGA was still high (82%) and decreased to <5% at 100 MBq/nmol. Under the same conditions, NO3S exhibited RCIs equal to 39 and 28%. Heating to 95 °C considerably increased the performances of both chelators, obtaining a quantitative RCI at 50 MBq/nmol and nearly identical labeling behavior (Figure 9D). Also at neutral pH, NO3S outperformed the results previously obtained with DO4S since the latter achieved quantitative incorporation at the maximal molar activity of 10 MBq/nmol at both RT and 95 °C.^26^

**Figure 10:**
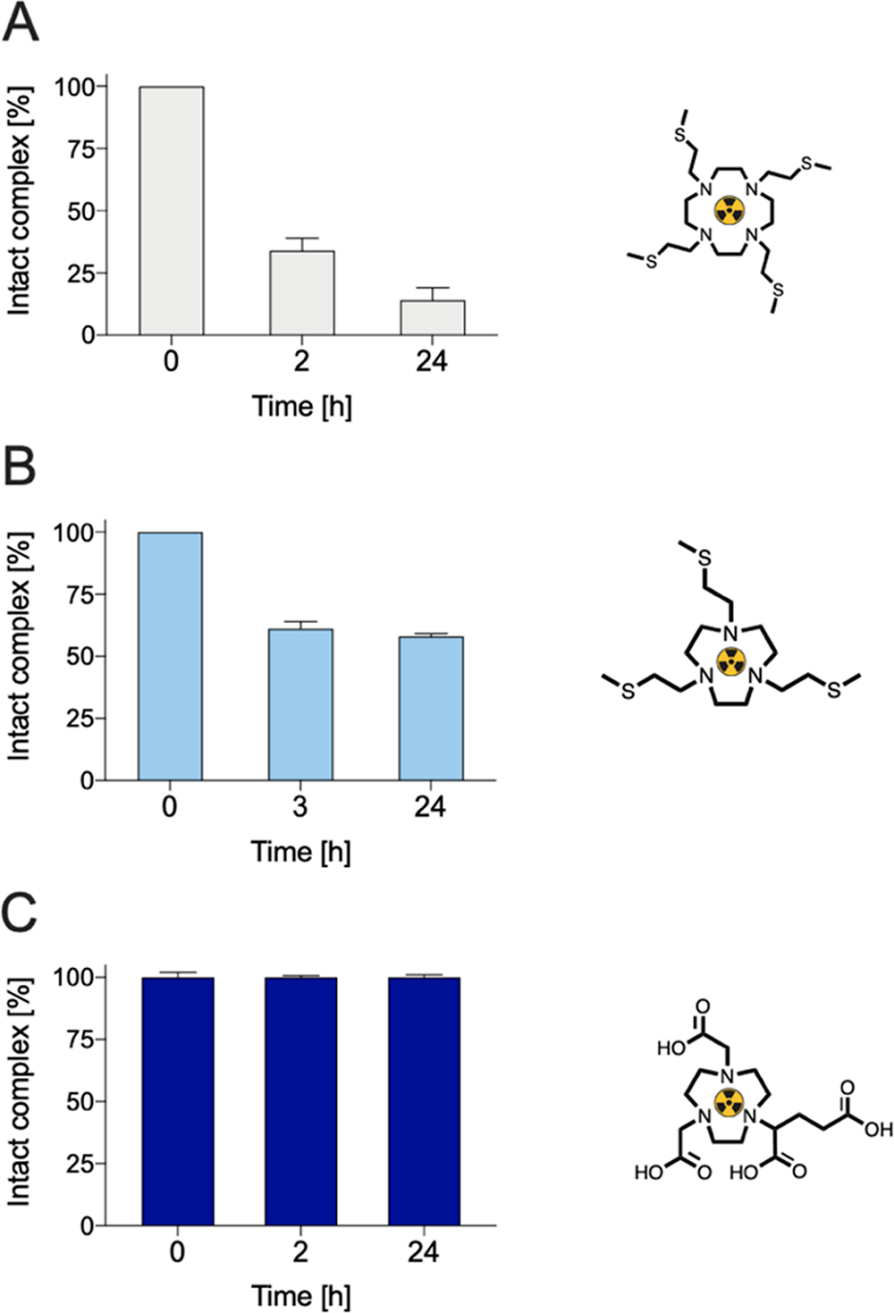
Integrity of (A) [^64^Cu][Cu(DO4S)]^2+^, (B) [^64^Cu][Cu(NO3S)]^2+^, and (C) [^64^Cu][Cu(NODAGA)]^−^ in human serum. Data reported in (A) were taken from our previous work.^26^

The human serum stability of [^64^Cu][Cu(NO3S)]^2+^ was evaluated to assess its integrity in the presence of biologically relevant ligands and metal ions that can respectively transchelate and transmetalate the complex *in vivo*. The obtained results, compared with those obtained with NODAGA and DO4S (data for the latter were taken from our previous work^26^), are shown in Figure 10.

[^64^Cu][Cu(NO3S)]^2+^ was demonstrated to be fairly labile in human serum since the percentage of [^64^Cu]Cu^2+^ bound to the chelator was around 60% after 24 h (Figure 10B), while DO4S formed a less-stable complex as its concentration dropped to 20% after the same time (Figure 10A). An even lower integrity was attained when the ring size was increased, passing from DO4S to TRI4S, as detailed in our previous work, attesting to NO3S as the most kinetically inert among all of the S-containing fully substituted polyazamacrocycles studied so far.^26^ When the results obtained with NO3S and DO4S are compared, it should be noted that the copper complex formed by DO4S is thermodynamically more stable than that formed by NO3S (Figure 5). However, herein we found that the latter is more kinetically inert in human serum. This enhanced inertness can be attributed to the saturated N_3_S_3_ coordination sphere around the metal center afforded by NO3S, which is likely beneficial to slowing down the complex disruption observed with DO4S.

Finally, the state-of-the-art chelator NODAGA afforded a more robust complex than NO3S, maintaining full integrity even at the later investigated time points (Figure 10C), still confirming the preference of Cu^2+^ for harder donor atoms such as oxygen. The origin of the lower stability of the [^64^Cu]Cu^2+^ complexes displayed in human serum by our sulfur-containing compounds is difficult to rationalize without dedicated experiments. On the basis of our CV results, we can exclude the occurrence of Cu^2+^/Cu^+^ reduction or other redox processes. Demetalation might be due to transchelation reactions occurring with proteins present in human serum with a high affinity for copper.

## Experimental Section

### General

Solvents and reagents were purchased from commercial suppliers and used without further purifications. 1,4,7-Triazacyclononane (TACN) and 1,4,7-triazacyclononane 1-glutaric acid-4,7-acetic acid (NODAGA) were purchased from Chematech. Ultrapure water (18.2 MΩ/cm, Purelab Chorus) was used throughout the work. Flash column chromatography was carried out using silica gel (60 Å, 230–400 mesh, 40–63 μm, Sigma-Aldrich) and proper mobile phases (*vide infra*). NMR spectra were collected on a 400 MHz Bruker Avance III HD spectrometer. Chemical shifts (δ) are reported in parts per million (ppm) and refer to either the residual solvent peak in organic solvents or 3-(trimethylsilyl)propionic acid sodium salt (TSP, 99%) in water. The coupling constants (*J*) are reported in hertz (Hz). Multiplicity is reported as follows: s, singlet; t, triplet; sx, sextet; m, multiplet; and br, broad peak. High-resolution electrospray ionization mass spectra (HR-ESI-MS) were recorded on an Agilent Technologies LC/MSD Trap SL mass spectrometer. Electronic spectra were recorded by using an Agilent Cary 60 UV–vis spectrophotometer. Radio-ultrahigh-performance liquid chromatography (radio-UHPLC) was performed on an Acquity system (Waters; Italy) equipped with a reversed-phase C18 column (1.7 μm, 2.1 mm × 150 mm), an Acquity Tunable UV–vis (TUV) detector (Waters; Milan, Italy), and a Herm LB 500 radiochemical detector (Berthold Technologies; Milan, Italy).

### Synthesis of the Chelators

#### 1,4,7-Tris[2-(methylsulfanyl)ethyl)]-1,4,7-triazacyclononane (NO3S)

TACN (0.690 g, 5.34 mmol, 1 equiv) and K_2_CO_3_ (4.925 g, 35.60 mmol, 6.6 equiv) were suspended in anhydrous acetonitrile (15 mL). 2-Chloroethyl methyl sulfide (2.11 mL, 21.4 mmol, 4 equiv) was slowly added under a N_2_ atmosphere. The reaction mixture was left to react at 40 °C for 24 h. After filtration and washing with acetonitrile (20 mL) and dichloromethane (20 mL), the solvent was removed under reduced pressure. The residue was purified by flash-column chromatography on silica gel using CH_2_Cl_2_/CH_3_OH/NH_4_OH 8:2:0.5 as the mobile phase to obtain NO3S as a dark-yellow oil (538.3 mg, yield 28.7%).^1^H NMR (400 MHz, CDCl_3_): δ 2.78 (s, 12, NCH_2_ ring), 2.74 (t, 6, NCH_2_ arms, *J* = 8.4 Hz), 2.57 (t, 6, SCH_2_, *J* = 8.6 Hz), 2.10 (s, 9, SCH_3_). ^13^C NMR (400 MHz, CDCl_3_): δ 55.68 (NCH_2_), 49.59 (NCH_2_), 29.57 (SCH_2_), 15.49 (SCH_3_). HR-ESI-MS: *m*/*z* [M + H]^+^: 352.2008 (found); 352.6375 (calcd for C_15_H_34_N_3_S_3_^+^).

#### 1,4,7-Tris-*n*-butyl-1,4,7-triazacyclononane (TACN-*n*-Bu)

TACN (0.11 g, 0.85 mmol, 1 equiv) and K_2_CO_3_ (0.47 g, 3.4 mmol, 4 equiv) were suspended in anhydrous acetonitrile (8 mL). Bromobutane (284 μL, 2.64 mmol, 3.1 equiv) was slowly added under a N_2_ atmosphere. The reaction mixture was left to react at 60 °C for 48 h. The solvent was removed under reduced pressure. The residue was purified by flash-column chromatography on silica gel using CH_2_Cl_2_/CH_3_OH 9:1 as the mobile phase to obtain TACN-*n*-Bu as a white oil (99.2 mg, 0.33 mmol, yield 39%).^1^H NMR (400 MHz, CDCl_3_): δ 3.14 (m br, 6, NCH_2_ ring), 2.99 (m br, 6, NCH_2_ ring), 2.82 (t, 6, NCH_2_ arms, *J* = 7.6 Hz), 1.54 (m, 6, CH_2_ arms), 1.35 (m, 6, CH_2_ arms), 0.95 (t, 9, CH_3_, *J* = 7.3 Hz). ^13^C NMR (400 MHz, CDCl_3_): δ 56.44 (NCH_2_), 50.94 (NCH_2_), 28.78 (CH_2_), 20.45 (CH_2_), 13.98 (CH_3_). HR-ESI-MS: *m*/*z* [M + H]^+^: 298.3236 (found); 298.3217 (calcd for C_18_H_40_N_3_^+^).

### Kinetic Experiments

The formation kinetics of Cu^2+^ complexes with NO3S and TACN-*n*-Bu was evaluated at different pH values at room temperature using UV–vis spectroscopy as previously reported.^24,25^ Briefly, equimolar amounts of Cu^2+^ and the proper ligands were mixed, and the corresponding UV–vis spectra were recorded over time. The final concentrations were *C*_Cu_^2+^ = *C*_L_ = 1.0 × 10^–4^ M. The pH was controlled using the following buffers: HCl 10^–2^ M (pH = 2), acetic acid/acetate (pH = 4), and 2-[4-(2-hydroxyethyl)piperazin-1-yl]ethanesulfonic acid (HEPES) (pH = 7.1).

### Thermodynamic Experiments

#### Protonation Constants

##### Potentiometry

Potentiometric titrations were conducted using an automated titrating system (Metrohm 765 Dosimat) equipped with a combined glass electrode (Hamilton pH 0–14) and a Metrohm 713 pH meter at *T* = 25 °C under a N_2_ atmosphere. NaNO_3_ (0.15 M) was used to fix the ionic strength. Stock solutions of NO3S and TACN-*n*-Bu were freshly prepared by dissolution of the synthesized compounds (∼10^–3^ M). To avoid carbonatation phenomena and facilitate the dissolution, prestandardized HNO_3_ (*C*_H_^+^ = 4*C*_L_) was coadded to each ligand solution. The solubility of the ligands in water depends on pH: minimal values occur at pH > 12, where the noncharged, totally deprotonated form predominates. All of the titrations were conducted as previously described.^22−25^ Potentiometric measurement demonstrated that the purity of the synthesized chelators was >95%. Each titration was performed independently, at least in quintuplicate.

##### NMR

^1^H NMR spectra of free NO3S and TACN-*n*-Bu (*C*_L_ = 1.0 × 10^–3^ M) were collected at *T* = 25 °C and *I* = 0.15 M NaNO_3_ at different pH in 90% H_2_O + 10% D_2_O. Small additions (∼μL) of HNO_3_ and/or NaOH were used to adjust the pH. The latter was measured as for the potentiometric measurements. Under highly acidic conditions, the pH was computed from the HNO_3_ concentration (pH = −log*C*_H_^+^). An excitation sculpting pulse scheme was used to suppress the water signal.^32^

#### Stability Constants of Cu^2+^ Complexes

UV–vis pH-spectrophotometric titrations were carried out as previously reported by means of the out-of-cell method at *T* = 25 °C and *I* = 0.15 M NaNO_3_.^24,25^ Briefly, stock solutions of NO3S or TACN-*n*-Bu were mixed with Cu(NO_3_)_2_ in independent vials at a 1:1 metal-to-ligand ratio (*C*_Cu_^2+^ = *C*_L_ = 1.0 × 10^–4^ M). The pH was adjusted as described in the NMR experiments. The vials were sealed and heated to *T* = 60 °C in a thermostatic bath to ensure complete complexation and then cooled to ambient temperature. The absorption spectra were recorded, and equilibrium was considered to be reached when no variations in either the pH or the electronic spectra were detected over time.

#### Stoichiometry of Cu^2+^ Complexes

The stoichiometry of the Cu^2+^ complex at pH = 7.1 was determined as described in our previous works.^24,25^ Briefly, solutions were prepared in which the metal-to-ligand ratio was varied at constant pH. To accurately determine the stoichiometry of the Cu^2+^-NO3S complex formed at basic pH (pH = 12), the Job method was applied, since under these conditions the similar absorbances of the complex and the free (excess) Cu^2+^ at λ = 280 nm make the previous method unfeasible. In this case, a series of independent solutions at different metal-to-ligand molar ratios were prepared, keeping the sum of their concentrations constant (*C*_Cu_^2+^ + *C*_NO3S_ = 2.0 × 10^–4^ M). The stoichiometry was determined by plotting the absorbance at the characteristic wavelength as a function of the metal-to-ligand ratio.

#### Data Processing

The thermodynamic data were elaborated with the least-squares fitting program PITMAP as described in our previous works.^22−25^ All equilibrium constants refer to the overall equilibrium *p*M^*m*+^ + *q*H^+^ + *r*L^*l*–^ ⇌ M_*p*_H_*q*_L_*r*_^*pm*+*q*–*rl*^, where M = Cu^2+^ and L = NO3S or TACN-*n*-Bu, respectively, and are defined as cumulative formation constants (log*β*_*pqr*_ = [M_*p*_L_*q*_H_*r*_]/[M]^*p*^[L]^*q*^[H]^*r*^).

### X-ray Crystallography

Crystals of NO3S suitable for X-ray diffraction were obtained in CHCl_3_ by the addition of NaPF_6_ in methanol after 24 h at 0 °C. Crystals of Cu^2+^-NO3S were prepared by mixing Cu(NO_3_)_2_·3H_2_O (27.8 mg, 0.115 mmol, 1 equiv) dissolved in water (2 mL) with NO3S (40.5 mg, 0.115 mmol, 1 equiv) dissolved in CHCl_3_ (2 mL). The solution was reacted for 30 min, filtered, and kept in a freezer for 2 days (−20 °C). The obtained crystals were washed with iced methanol (4 mL) (yield 43.7%). X-ray measurements were conducted at room temperature on a Nicolet P3 instrument using a numerical absorption correction with graphite monochromate Mo Kα radiation as previously described.^24^ The SHELX 93 crystallographic software package was used.^33^ The graphical representation and the edition of CIF files were created with Mercury software.^34^ The structures were deposited with CCDC numbers of 2067485 for [H(NO3S)]PF_6_ and 2067484 for [Cu(NO3S)][Cu(NO_3_)_4_].

### Cyclic Voltammetry and Electrolysis

Cyclic voltammetry (CV) experiments were carried out as reported in our former works.^24,25^ Briefly, a six-necked cell equipped with three electrodes and connected to an Autolab PGSTAT 302N potentiostat interfaced with NOVA 2.1 software (Metrohm) was used. A glassy-carbon working electrode (WE) fabricated from a 3-mm-diameter rod (Tokai GC-20), a platinum wire as a counter electrode (CE), and a saturated calomel electrode (SCE) as the reference electrode (RE) were employed. All CVs were performed at ambient temperature in aqueous 0.15 M NaNO_3_ at *C*_Cu_^2+^ = *C*_NO3S_ = 1.0 × 10^–3^ M. The pH of the solutions was adjusted with small aliquots (μL) of NaOH and/or HNO_3_ solutions. CV with scan rates ranging from 0.005 to 0.2 V/s was recorded in the region from −0.5 to 0.5 V. At this potential range, the solvent with the supporting electrolyte and the free ligand were found to be electroinactive.

Bulk electrolysis of the preformed Cu^2+^-NO3S complexes was carried out in a two-compartment cell. The WE was a large-area glassy carbon, and the CE was a Pt gauze in 0.15 M NaNO_3_, separated from the WE solution by two glass frits (G3). The RE was SCE. The electrolysis was performed at a fixed potential equal to *E* = −0.35 V and monitored by linear scan voltammetry on a rotating disk electrode. The electrolysis was considered to be complete when the cathodic current reached 2% of the initial value. At the end of each electrolysis, the solution was transferred to the NMR tube under an inert atmosphere (Ar). After the transfer, the atmosphere was filled with inert gas. ^1^H NMR spectra of the *in situ*-generated Cu^+^ complexes at different pH values were collected at *T* = 25 °C as detailed in the previous section. No variations of the ^1^H NMR spectra were detected after 2 days (tubes were stored under a normal atmosphere). The stability constant of the Cu^+^-NO3S complex was obtained as described in our previous work.^24^

### [^64^Cu]Cu^2+^ Radiolabeling and Human Serum Integrity

***Caution!** [*^*64*^*Cu]Cu*^*2*+^*is a radionuclide that emits ionizing radiation, and it was manipulated in a specifically designed facility under appropriate safety controls*.

#### Radiolabeling

[^64^Cu]CuCl_2_ in 0.5 M HCl (molar activity around 40 GBq/nmol) was purchased from Advanced Center Oncology Macerata ACOM (Italy). NO3S and NODAGA stock solutions were prepared in ultrapure water at 1.0 × 10^–3^ M and diluted appropriately to give a serial dilution series (1.0 × 10^–4^–1.0 × 10^–7^ M).

Radiolabeling data were collected through the addition of [^64^Cu]CuCl_2_ diluted in 0.05 M HCl (1 MBq, 10 μL) to a solution containing the ligand (20 μL) diluted in sodium acetate buffer (90 μL, pH = 4.5) or sodium phosphate buffer (90 μL, pH = 7). Different apparent molar activities were tested from 0.1 to 1000 MBq/nmol, corresponding to a final ligand concentration ranging from 1.0 × 10^–4^ to 1.0 × 10^–9^ M. The reaction mixtures were allowed to react for 10 min at ambient temperature or 95 °C. All radiolabeling reactions were repeated at least in triplicate. Radiochemical incorporation (RCI) was determined via radio-UHPLC using the analytical method previously described.^26^ Alternatively, radio-thin layer chromatography (radio-TLC) on silica gel 60 RP-18 F254S plates with sodium citrate (1 M, pH = 4) as the eluent was employed. Under these conditions, free [^64^Cu]Cu^2+^ migrates with the solvent front (*R*_f_ = 1) while [^64^Cu]Cu^2+^ complexes remain at the baseline (*R*_f_ = 0). A Cyclone Plus Storage Phosphor System (PerkinElmer) was used to analyze the radio-TLC plates after their exposure to a super-resolution phosphor screen (type MS, PerkinElmer; Waltham, MA, USA). All of the data were processed with OptiQuant software (version 5.0, PerkinElmer Inc.; Waltham, MA, USA).

#### Human Serum Integrity

The integrity of [^64^Cu][Cu(NO3S)]^2+^ and [^64^Cu][Cu(NODAGA)]^−^ (as controls) was assessed over time by incubation of the preformed complexes (prepared using the radiolabeling protocol described above) in human serum at *T* = 37 °C (1:1 *V*/*V* dilution). The radiometal-complex stability was monitored at different time points over the course of 24 h via radio-TLC following the protocol reported for radiolabeling studies.

## Conclusions

The biologically triggered reduction of Cu^2+^ to Cu^+^ has been postulated as a possible demetalation pathway in ^64/67^Cu-based radiopharmaceuticals. To hinder this phenomenon, we have previously developed a family of N- and S-containing macrocycles capable of efficiently accommodating both oxidation states. Unfortunately, under highly dilute radiochemical conditions, a marked radiometal release was observed in human serum likely because of the partially saturated coordination sphere around the metal center. In this work, we have hypothesized that switching to a smaller macrocyclic backbone could avoid the hitherto observed demetalation by fully encapsulating and saturating the coordination sphere of the copper ion. For this purpose, the new hexadentate macrocyclic ligand NO3S was synthesized by introducing three sulfanyl pendants on a TACN backbone.

While conserving a high thermodynamic stability for both copper oxidation states, NO3S proved to be superior to the previously studied full S-substituted chelators in the radiolabeling performances with [^64^Cu]Cu^2+^ and in the human serum integrity. These findings suggest that a copper ion fully encapsulated in a N_3_S_3_ hexacoordinate environment as in [Cu(NO3S)]^2+^ could afford a more inert complex *in vivo* than Cu complexes possessing a N_4_ + N_4_S_ax_ or N_4_S_ax_ coordination sphere such as [Cu(DO4S)]^2+^. The results obtained herein prove that the TACN ring is a more promising backbone (with respect to cyclen and to larger rings) to build up new copper S-containing chelators. The overall lower performances evidenced by NO3S when compared to NODAGA and the lower stability of the Cu^2+^-NO3S complexes when compared to the Cu^2+^-NOTA complexes suggest that the presence of oxygen donors appended on the TACN backbone is beneficial to the formation of stable Cu^2+^ complexes, but at least one sulfur atom might likely be essential to stabilizing them upon *in vivo* reduction to Cu^+^. Hence, the development and study of hybrid sulfur-carboxylic TACN derivatives might be a useful strategy to fulfill all of the requested demands.

## Supporting Information Available

Figures of other chelators used in copper-based radiopharmaceuticals; schemes of the syntheses of NO3S and TACN-*n*-Bu; corresponding NMR and ESI-MS spectra; NMR and UV–vis spectra of the free ligands and of the metal–ligand complexes at various pH values (tables and figures) and corresponding fitting lines where applicable; voltammetric data; crystallographic data; and a representative HPLC radio chromatogram (PDF)
