# Supplementary material for: Is Smaller Better? Cu2+/Cu+ Coordination Chemistry and Copper-64 Radiochemical Investigation of a 1,4,7-Triazacyclononane-Based Sulfur-Rich Chelator
Source: Inorg Chem. 2023 Apr 28;62(50):20621–33. doi: 10.1021/acs.inorgchem.3c00621 (PMC10731632; doi:10.1021/acs.inorgchem.3c00621)
Supplement: Supplementary file 1 — ic3c00621_si_001.pdf [file ic3c00621_si_001.pdf]

Supporting Information

Is Smaller Better? Cu^{2+}/Cu^{+} Coordination Chemistry and Copper-64 Radiochemical Investigation of a 1,4,7-Triazacyclononane-Based Sulfur-Rich Chelator

Marianna Tosato[1], Sara Franchi[1], Abdirisak Ahmed Isse[1], Alessandro Del Vecchio[1], Giordano Zanoni[1], André Alker[2], Mattia Asti[3], Thomas Gyr[4], Valerio Di Marco[1,*], Helmut Mäcke[5,*]

[1] Department of Chemical Sciences, University of Padova, 35131 Padova, Italy

[2] Roche Pharmaceutical Research and Early Development, Roche Innovation Center Basel F. Hoffmann-La Roche, 4058 Basel, Switzerland

[3] Radiopharmaceutical Chemistry Section, Nuclear Medicine Unit, AUSL-IRCCS Reggio Emilia, 42122 Reggio Emilia, Italy

[4] Division of Radiopharmaceutical Chemistry, Clinic of Radiology and Nuclear Medicine, University Hospital Basel, 4031 Basel, Switzerland

[5] Department of Nuclear Medicine, University Hospital Freiburg, D-79106 Freiburg, Germany

*** Corresponding authors**: valerio.dimarco@unipd.it, helmut.maecke@uniklinik-freiburg.de

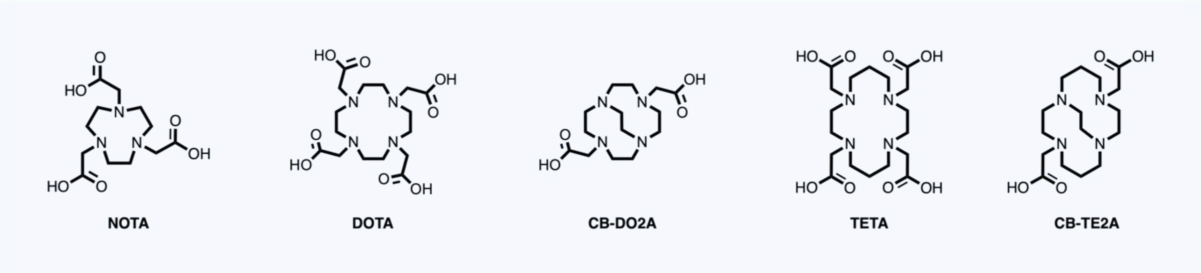

Figure S1. Representative state-of-the-art ligands for [$^{64/67}$Cu]Cu^{2+}.

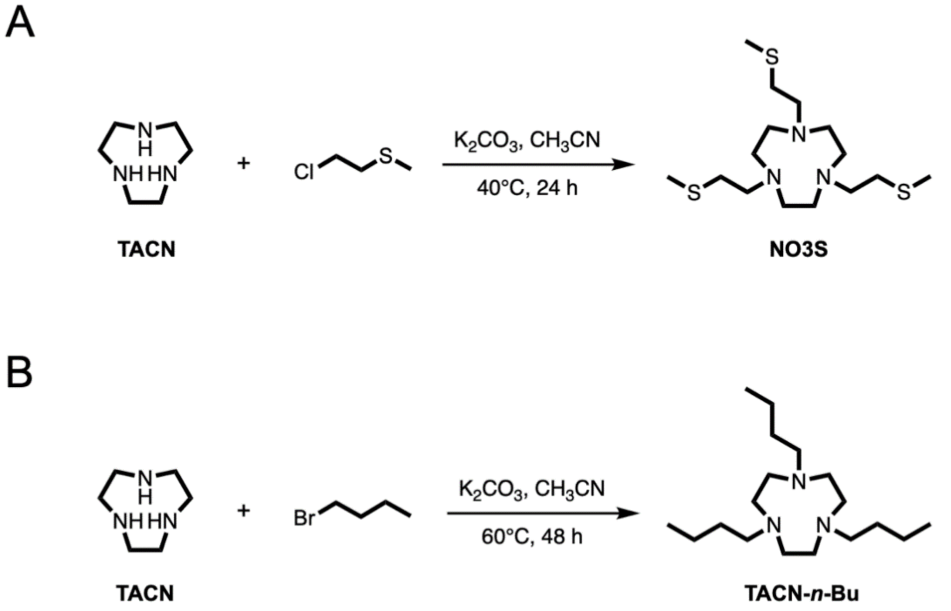

Figure S2. Synthesis of (A) NO3S and (B) TACN-*n*-Bu.

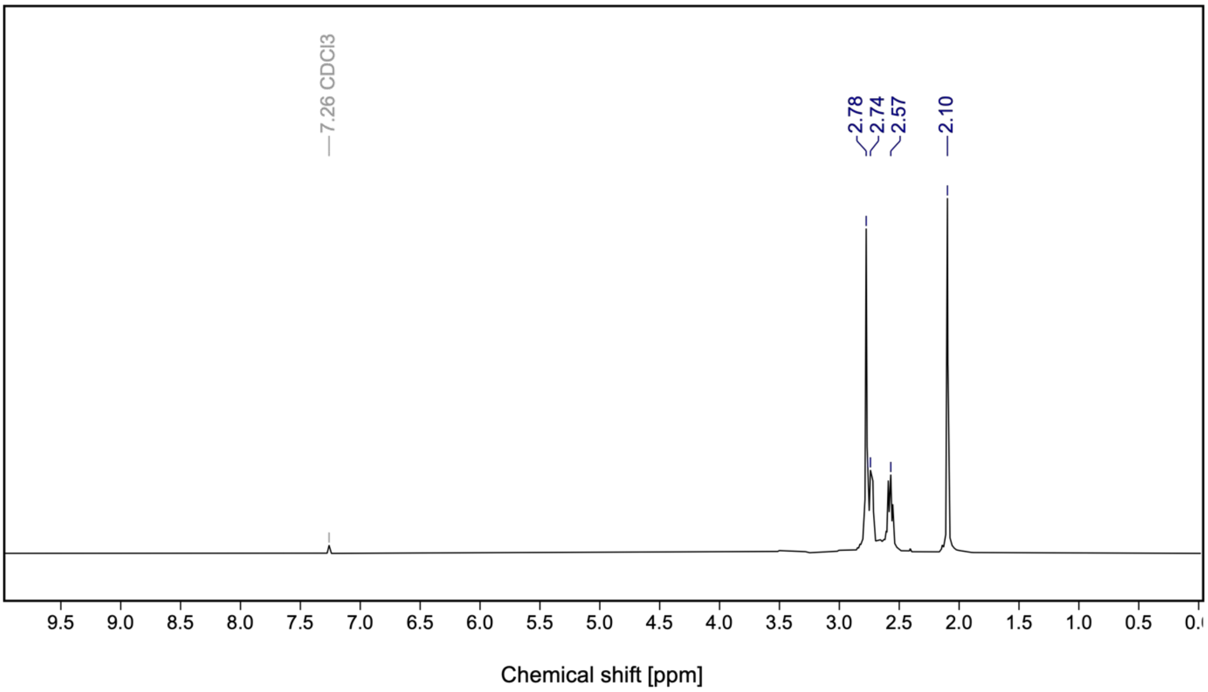

Figure S3. ^{1}H-NMR spectrum ($CDCl_3$, 400 MHz, T = 25 °C) of NO3S.

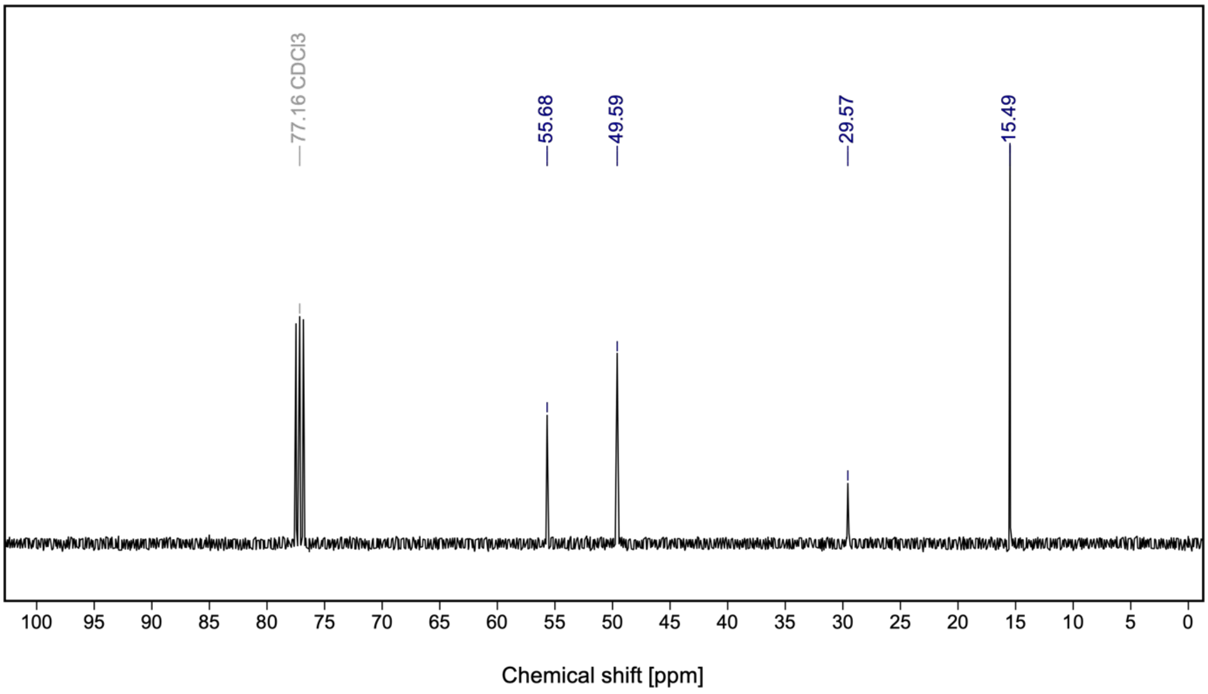

Figure S4. $^{13}C\{^{1}H\}$-NMR spectrum ($CDCl_3$, 400 MHz, T = 25 °C) of NO3S.

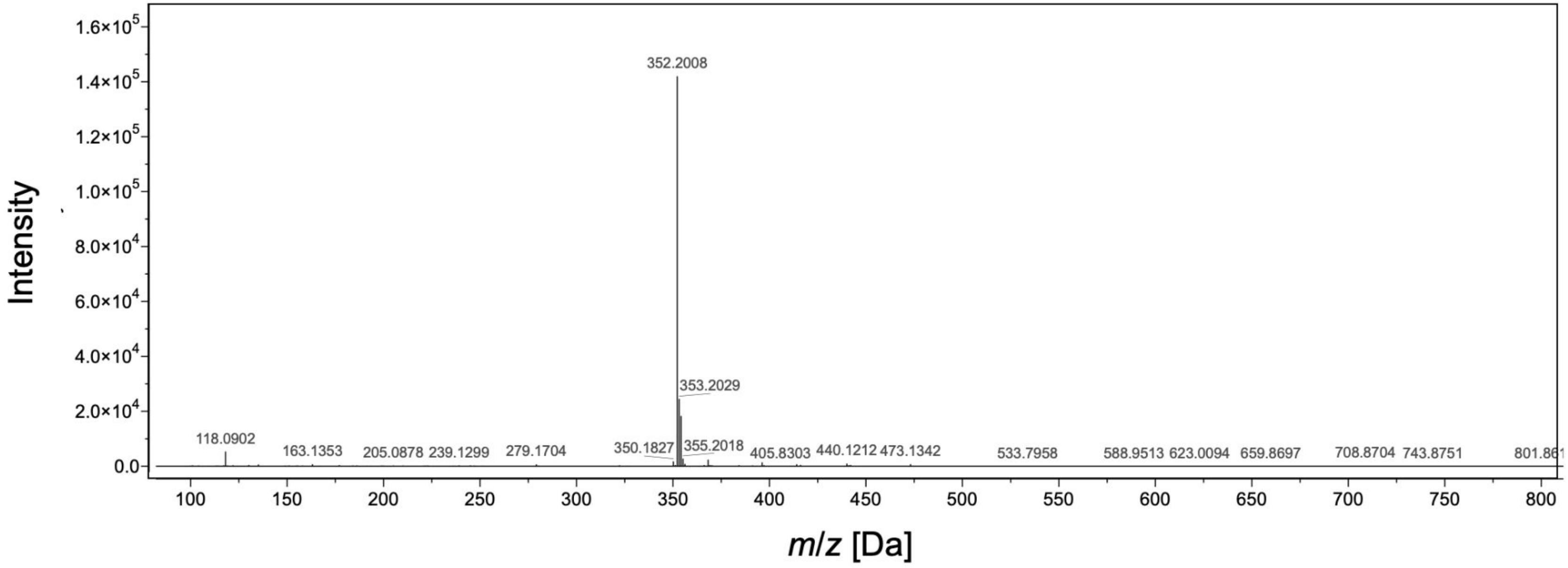

Figure S5. HR-ESI-MS spectrum of NO3S.

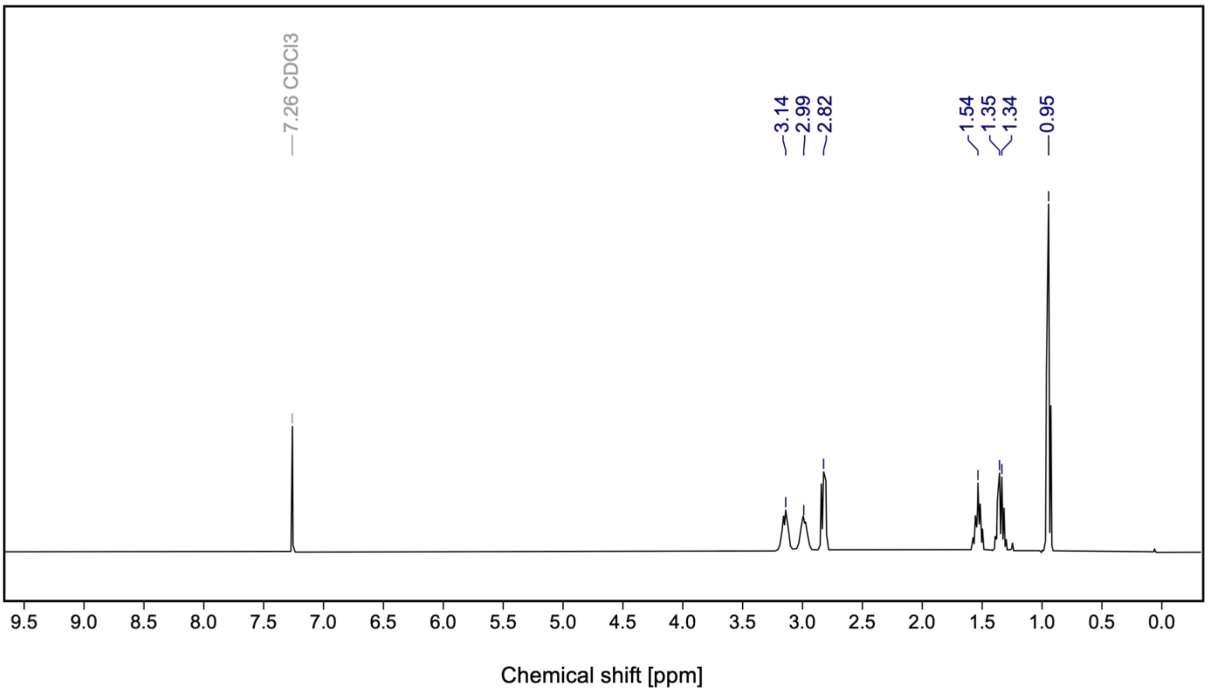

Figure S6. ^{1}H-NMR spectrum (400 MHz, T = 25 °C, $CDCl_3$) of TACN-*n*-Bu.

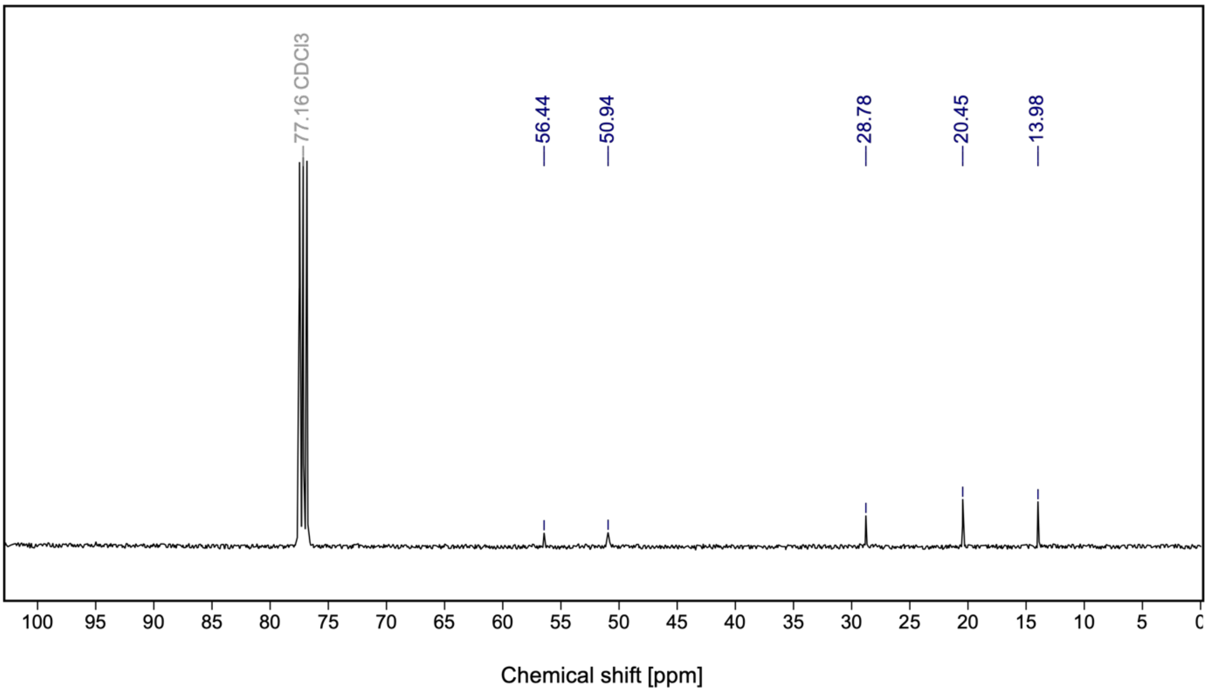

Figure S7. ^{13}C{^{1}H}-NMR spectrum (400 MHz, T = 25 °C, $CDCl_3$) of TACN-*n*-Bu.

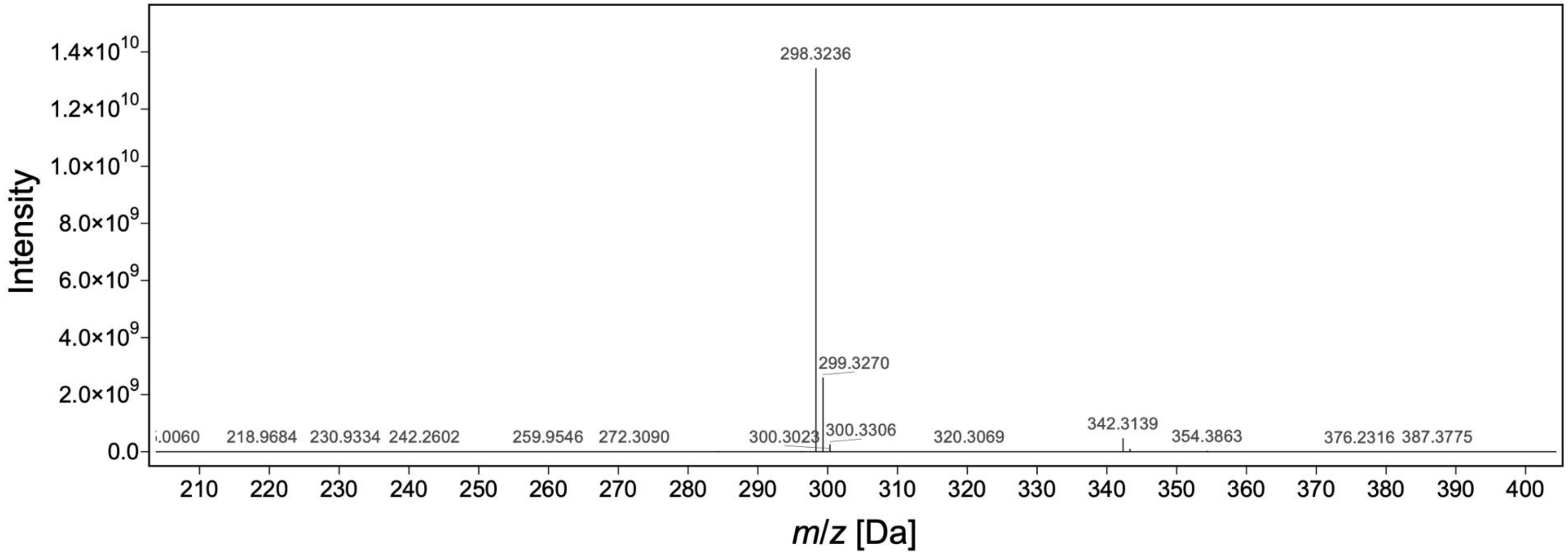

Figure S8. HR-ESI-MS spectrum of TACN-*n*-Bu.

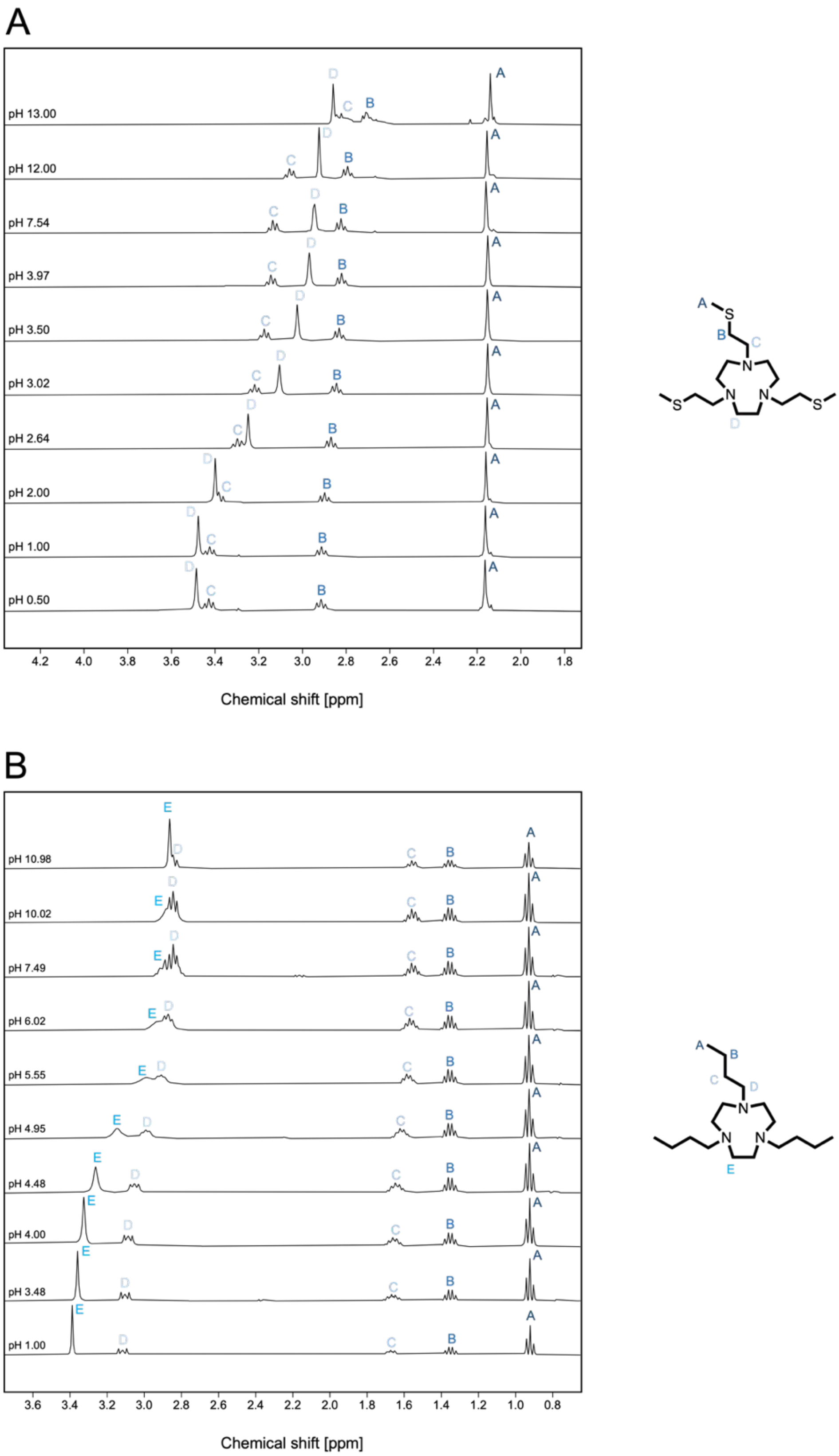

Figure S9. ^{1}H-NMR spectra (400 MHz, 90% H_2O + 10% D_2O, I = 0.15 M $NaNO_3$, T = 25 °C) at different pH of (A) NO3S (C_{NO3S} = $1.0 \cdot 10^{-3}$ M) and (B) TACN-*n*-Bu ($C_{TACN\text{-}n\text{-}Bu}$ = $1.0 \cdot 10^{-3}$ M) and signal attributions. Precipitates were observed at pH > 12 for NO3S and pH > 10.8 for TACN-*n*-Bu.

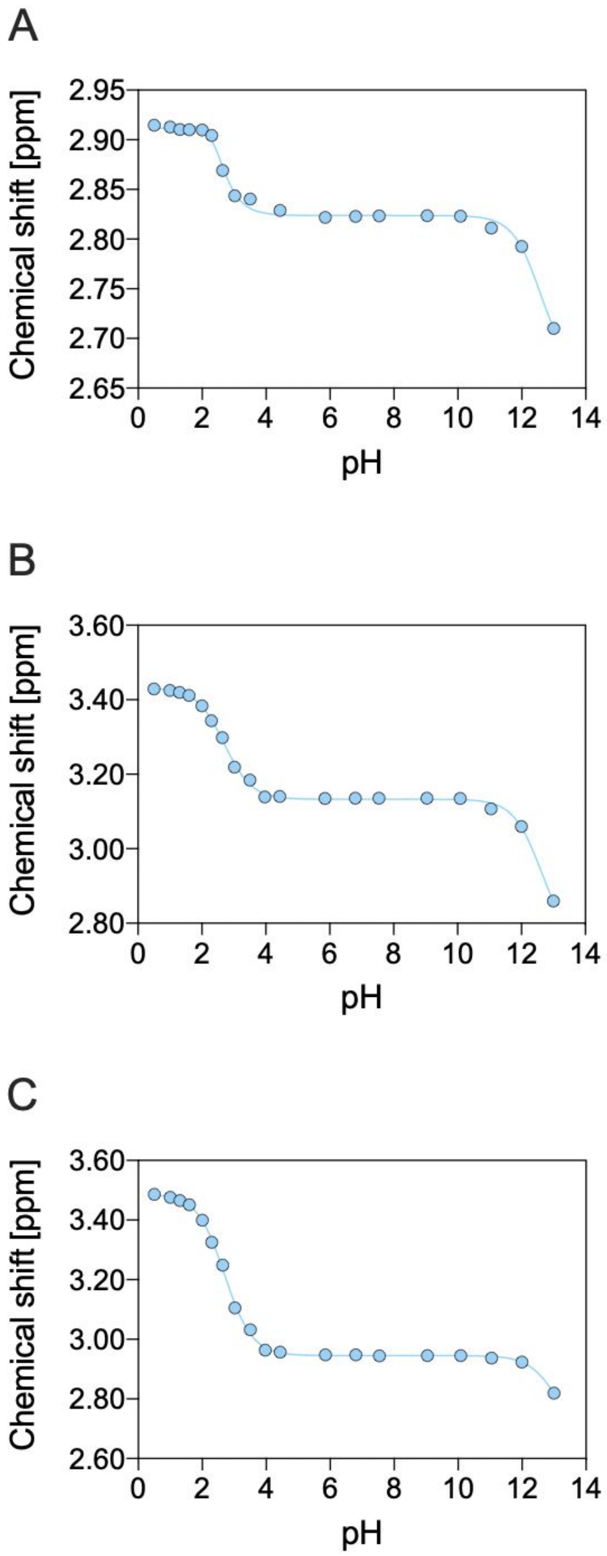

Figure S10. Representative variation of the chemical shift of (A) SCH_2, (B) NCH_2 arms and (C) NCH_2 ring protons as a function of pH for NO3S (data points were taken from **Figure S9** - **A**) and corresponding fitting lines.

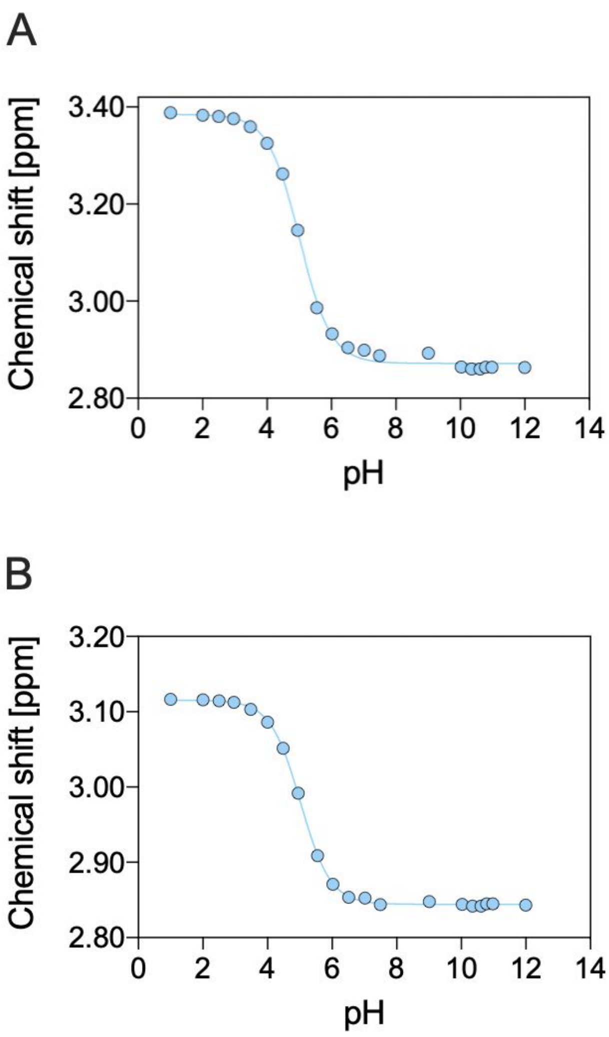

Figure S11. Representative variation of the chemical shift of (A) NCH_2 ring and (B) NCH_2 arms protons as a function of pH for TACN-*n*-Bu (data points were taken from **Figure S9** - **B**) and corresponding fitting lines.

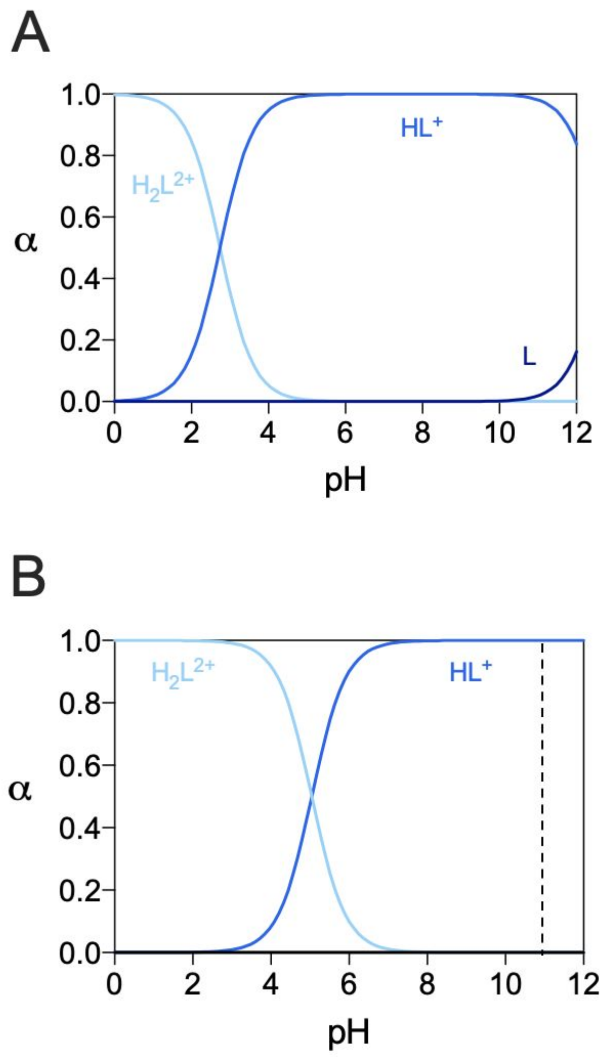

Figure S12. Distribution diagram of (A) NO3S and (B) TACN-*n*-Bu. The dashed line indicates the formation of sparingly soluble L.

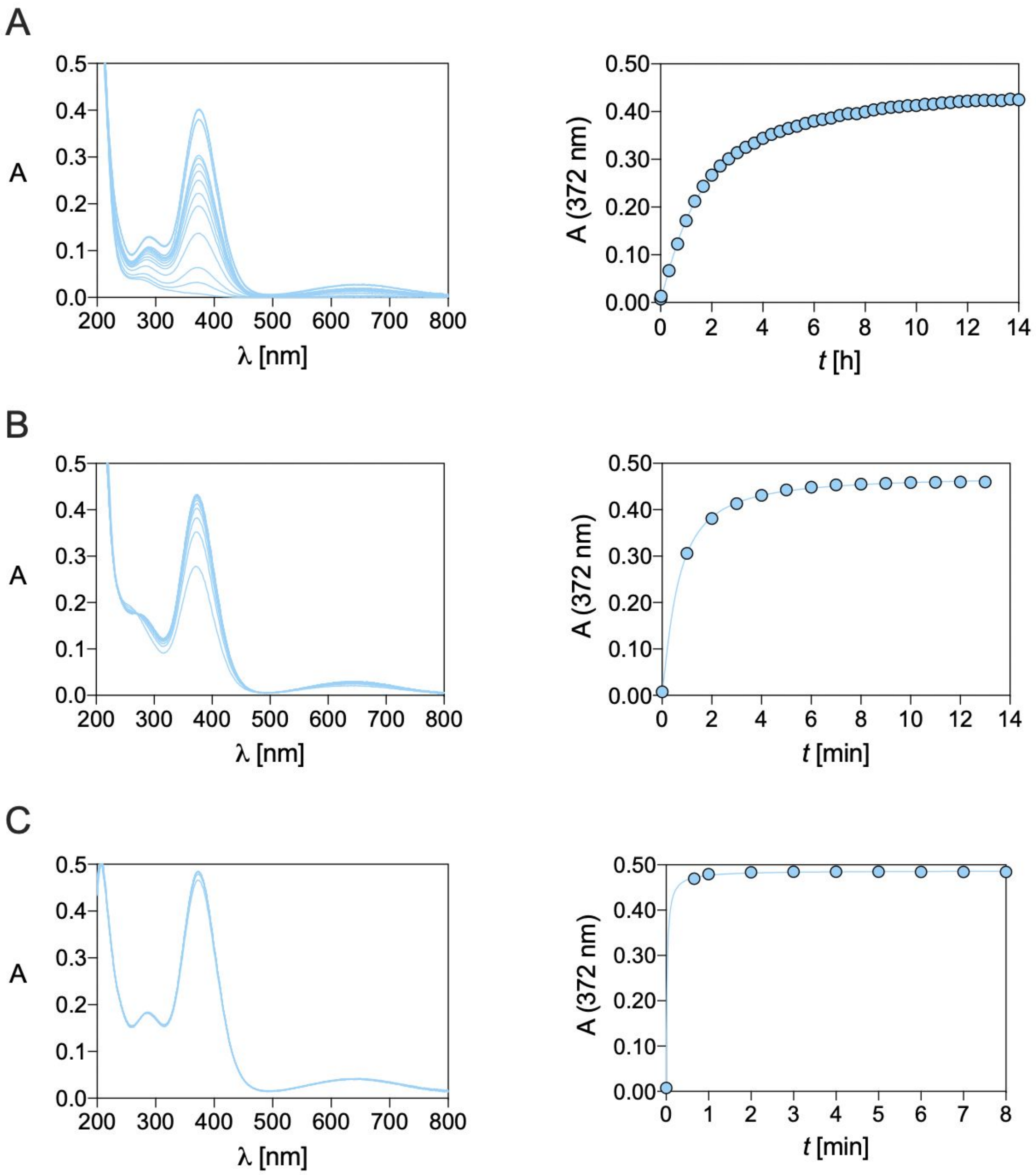

Figure S13. UV-Vis spectra (left) and trend of absorbance at 372 nm *vs.* time (right) relative to the Cu^{2+}-NO3S complex formation at (A) pH 1.0, (B) pH 4.0 and (C) pH 7.1 ($C_{NO3S} = C_{Cu^{2+}} = 1.0 \cdot 10^{-4}$ M, ambient temperature). The absorbance of the ligand at 372 nm prior to the addition of Cu^{2+} was included in the graphs reported on the right to represent the $t = 0$ point.

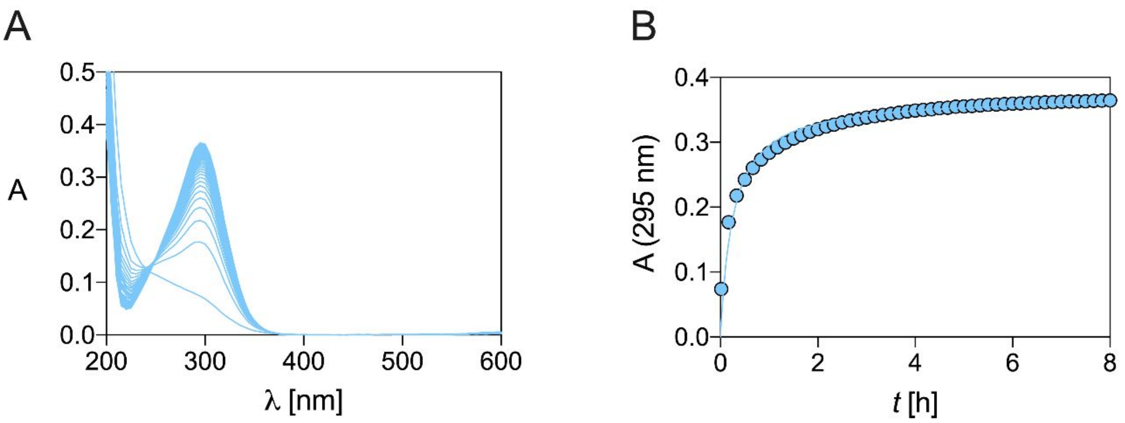

Figure S14. UV-Vis spectra (left) and trend of absorbance at 295 nm *vs.* time (right) relative to the Cu^{2+}-TACN-*n*-Bu complex formation at pH 7.1 ($C_{TACN\text{-}n\text{-}Bu} = C_{Cu^{2+}} = 1.0 \cdot 10^{-4}$ M, ambient temperature).

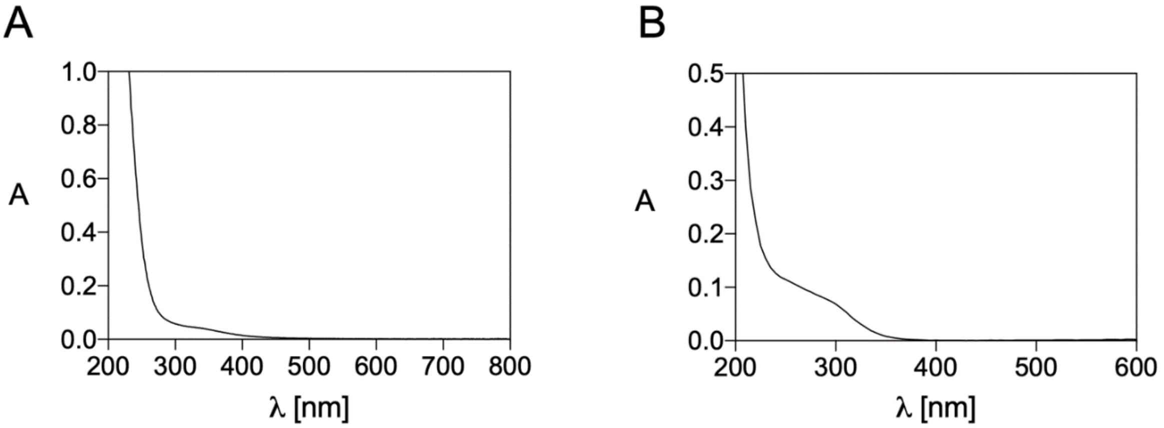

Figure S15. Representative UV-Vis spectra of (a) NO3S ($C_{NO3S} = 1.0 \cdot 10^{-4}$ M, pH 4.5) and (B) TACN-*n*-Bu ($C_{TACN\text{-}n\text{-}Bu} = 1.0 \cdot 10^{-4}$ M, pH 7.1) in aqueous solution and ambient temperature.

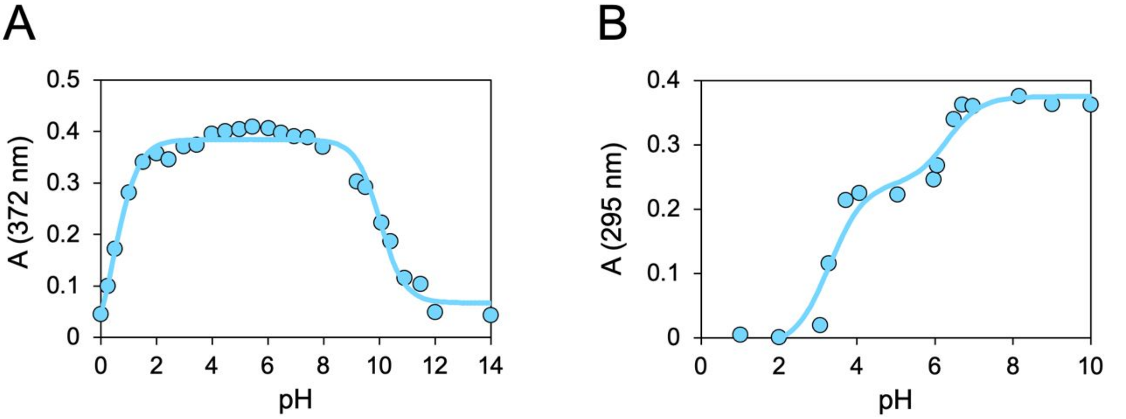

Figure S16. Variation of the absorbance of the Cu^{2+} complexes as a function of pH for (A) NO3S at 372 nm and (B) TACN-*n*-Bu at 295 nm. Data points were taken from **Figure 3**. The lines represent the fitting curves which allowed to determine the stability constants.

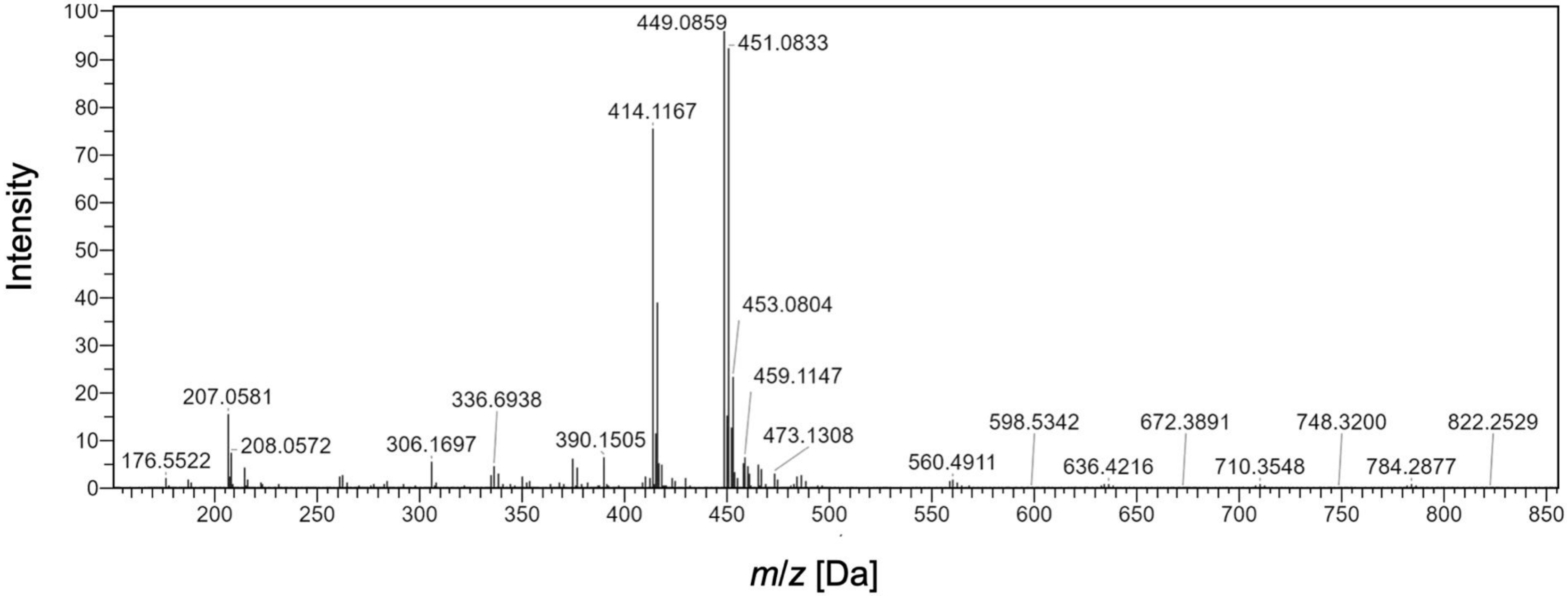

Figure S17. HR-ESI-MS spectrum of $[Cu(NO3S)]^{2+}$ (pH 4): the fragment at *m*/*z* = 449.0859/451.0833 corresponds to $[Cu(NO3S)Cl]^{+}$ while the fragment at *m*/*z* = 414.1167 to $[Cu(NO3S)]^{+}$.

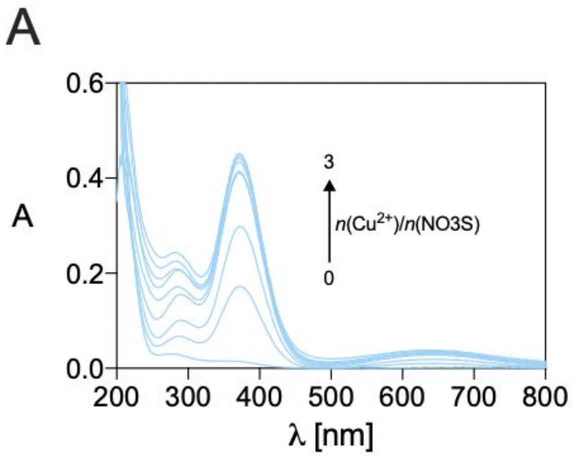

Figure S18. (A) UV-Vis spectrophotometric titrations at different metal-to-ligand molar ratio of Cu^{2+}-NO3S (C_{NO3S} = 1.0·10^{-4} M) at pH 7.1 and T = 25 °C and (B) absorbance at λ_{max} = 372 nm *vs.* $n(Cu^{2+})/n$(NO3S).

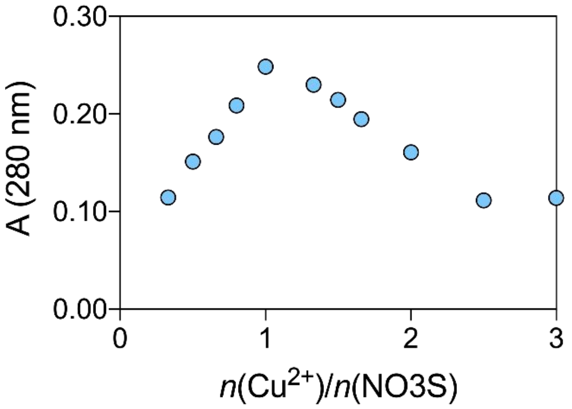

Figure S19. Absorbance at λ = 280 nm *vs*. molar ratio $n(Cu^{2+})/n$(NO3S) at ($C_{Cu^{2+}}$ + C_{NO3S}) = 2.0·10^{-4} M, pH 12 and T = 25 °C (Job method).

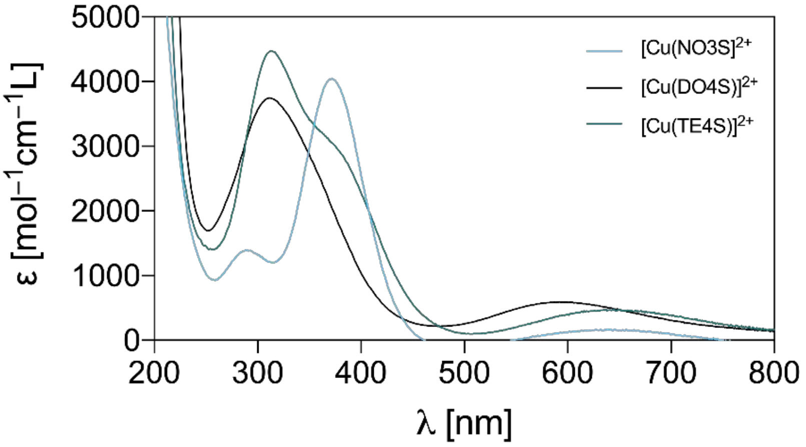

Figure S20. Comparison of the normalized UV-Vis spectra of $[Cu(NO3S)]^{2+}$, $[Cu(DO4S)]^{2+}$ and $[Cu(TE4S)]^{2+}$. Data for $[Cu(DO4S)]^{2+}$ and $[Cu(TE4S)]^{2+}$ were taken from our previous works.[1,2]

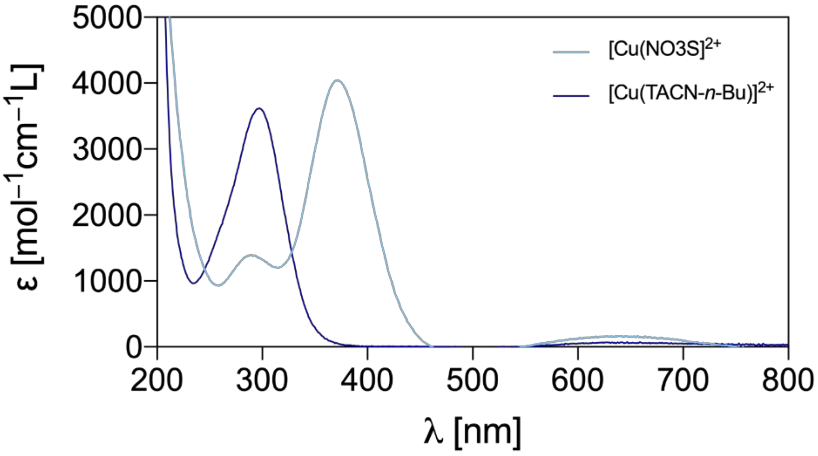

Figure S21. Comparison of the normalized UV-Vis spectra of $[Cu(NO3S)]^{2+}$ and $[Cu(TACN\text{-}n\text{-}Bu)]^{2+}$.

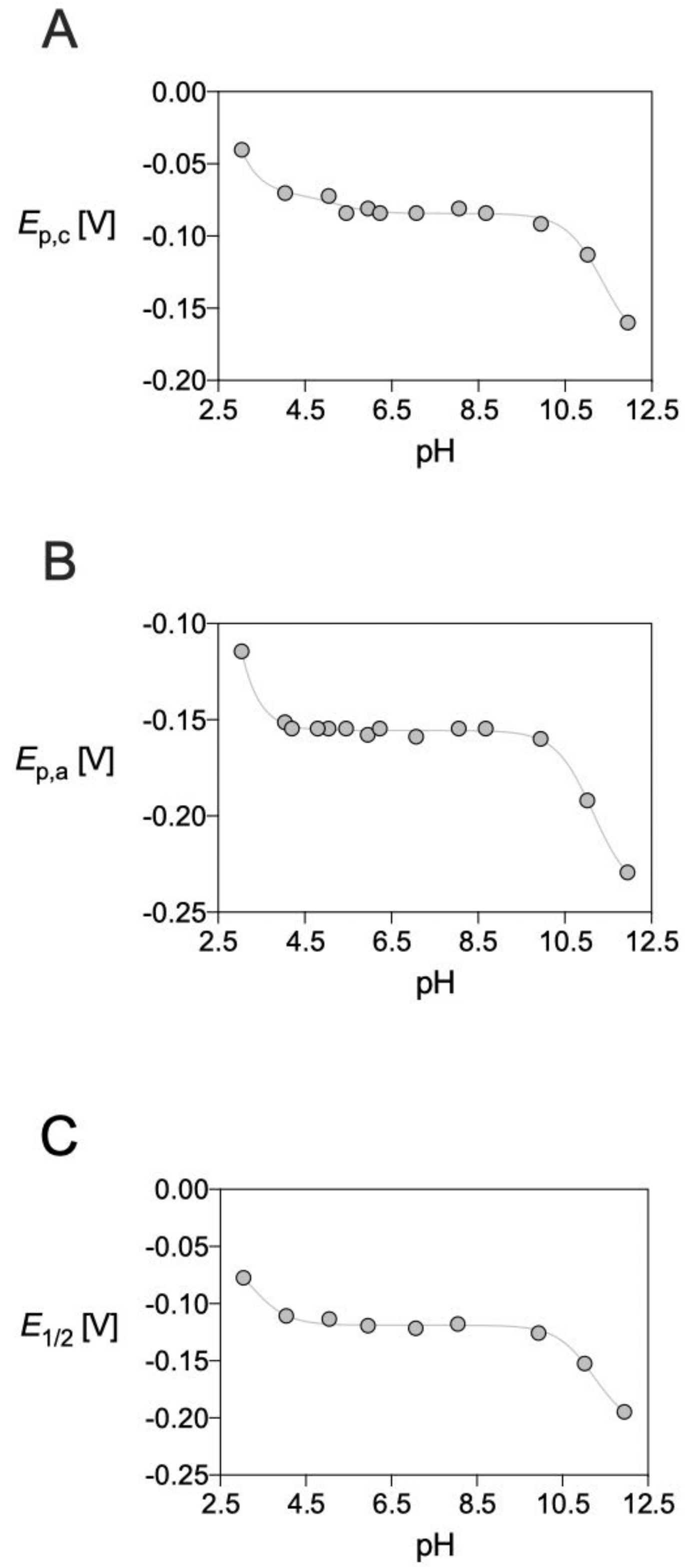

Figure S22. Variation of the (A) cathodic peak potential, (B) anodic peak potential and (C) $E_{1/2}$ of the $[Cu(NO3S)]^{2+}/[Cu(NO3S)]^{+}$ couple as a function of pH.

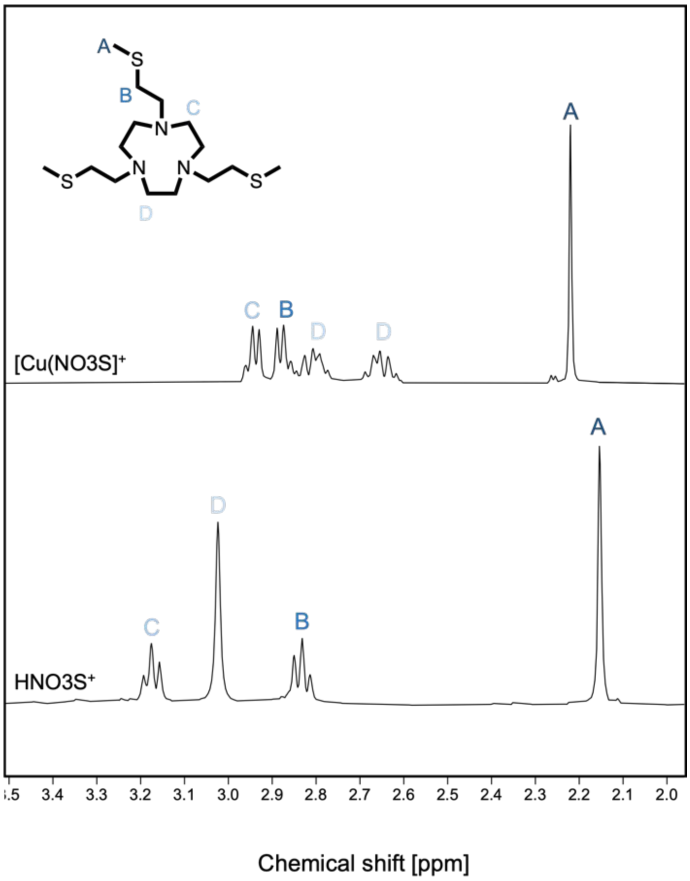

Figure S23. Comparison of the ^{1}H-NMR spectra (400 MHz, 90% H_2O + 10% D_2O, T = 25 °C) of the *in situ* generated Cu^+ complex of NO3S and the free monoprotonated ligand (pH 7, I = 0.15 M $NaNO_3$, $C_{[Cu(NO3S)]^+}$ or C_{HNO3S^+} = $1.0 \cdot 10^{-3}$ M).

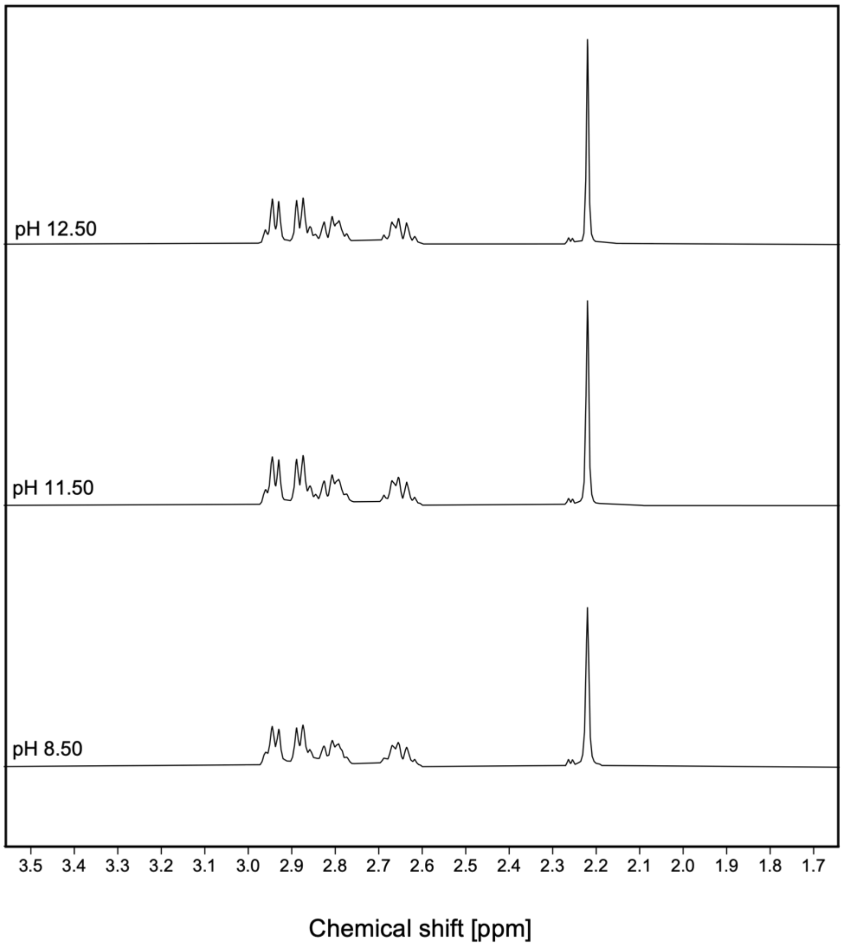

Figure S24. ^{1}H NMR spectra (400 MHz, 90% H_2O + 10% D_2O, I = 0.15 M $NaNO_3$, T = 25 °C) of the *in situ* generated Cu^+-NO3S complex at different pH ($C_{[Cu(NO3S)]^+}$ = $1.0 \cdot 10^{-3}$ M).

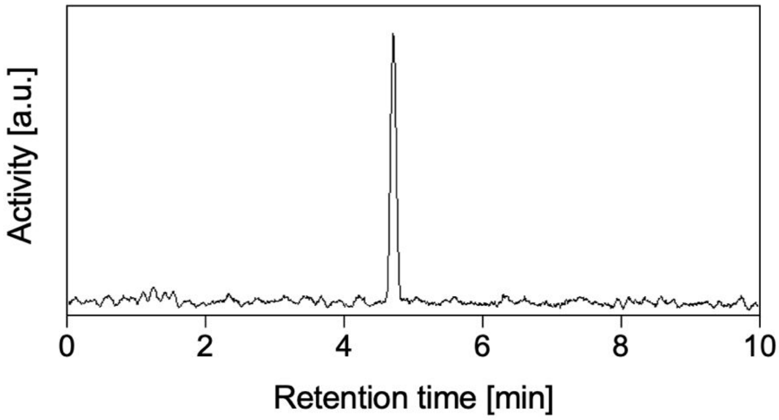

Figure S25. Representative HPLC radio-chromatogram of $[^{64}Cu][Cu(NO3S)]^{2+}$ (t_R = 4.7 min).

Table S1. Chemical shifts, multiplicities, relative integral, and proton attribution for the differently protonated forms of NO3S. Chemical shift values in 90% H_2O + 10% D_2O differ from those in $CDCl_3$ due to the solvent effects and the different protonation states of the ligand. Data were taken from **Figure S9 - A**. L represents the completely deprotonated form shown in **Figure 1 - C**.

Species	Chemical shift [ppm]	Multiplicity	Relative integral	Proton Attribution
L	2.16	s	9	SCH_3
	2.71 - 2.82	t	6	SCH_2
	2.82 - 2.94	m	12	NCH_2 ring
	2-85 - 3.13	t	6	NCH_2 side arms
HL^+	2.16	s	9	SCH_3
	2.81 - 2.90	t	6	SCH_2
	2.93 - 3.32	s	12	NCH_2 ring
	3.13 - 3.34	t	6	NCH_2 side arms
H_2L^{2+}	2.16	s	9	SCH_3
	2.84 - 2.91	t	6	SCH_2
	3.21 - 3.42	t	6	NCH_2 side arms
	3.13 - 3.34	s	12	NCH_2 ring

s = singlet, t = triplet, m = multiplet

Table S2. Chemical shifts, multiplicities, relative integral, and proton attribution for the differently protonated forms of TACN-*n*-Bu. Chemical shift values in 90% H_2O + 10% D_2O differ from those in $CDCl_3$ due to the solvent effects and the different protonation states of the ligand. Data were taken from **Figure S9 - B**. L represents the completely deprotonated form shown in **Figure 1 - C**.

Species	Chemical shift [ppm]	Multiplicity	Relative integral	Proton Attribution
HL^+	0.93	t	9	CH_3
	1.36	sx	6	$-CH_2\underline{CH_2}CH_3$
	1.56	q	6	$-\underline{CH_2}CH_2CH_3$
	2.74 - 2.98	m	18	NCH_2
H_2L^{2+}	0.92	t	9	CH_3
	1.36	sx	6	$-CH_2\underline{CH_2}CH_3$
	1.67	q	6	$-\underline{CH_2}CH_2CH_3$
	3.12	t	6	NCH_2 side arms
	3.38	s	12	NCH_2 ring

s = singlet, t = triplet, q = quintet, sx = sextuplet

Table S3. Crystallographic data and refinement details for [H(NO3S)](PF_6).

Empirical formula	$C_{15}H_{34}F_6N_3PS_3$
Formula weight	494.58 g/mol
Temperature	183 (2)
Radiation and wavelength	Mo-Kα, λ = 1.54178 Å
Crystal system	Monoclinic
Space group	P2 (1)/n
Unit cell dimension	*a* = 10.175(10) Å α = 90.0°<br>*b* = 17.076(2) Å β = 94.080(10)°<br>*c* = 13.443(10) Å, γ = 90.0°
Volume	2329.8 $Å^3$
Z	4
Density (calculated)	1.410 Mg/m^3
Absorption coefficient, μ	4.063 mm^{-1}
F(000)	1036
Crystal size	0.4 × 0.4 × 0.3 mm
θ area data collection	5.09 to 50.00°
Index area	$0 \le h \le 11$, $-1 \le k \le 18$, $-14 \le l \le 14$
Reflections collected	2512
Independent reflections	2316 [*R*(int) = 0.0597]
Refinement method	Full-matrix least squares on F^2
Data/restraints/parameters	2304/0/273
Final R indices [I <2σ(I)]	R_1 = 0.0740, wR_2 = 0.2076
R indices (all data)	R_1 = 0.0853, wR_2 = 0.2365
Extinction coefficient	0.0020(6)
Differential signals	0.678 and −0.43 e.$Å^{-3}$

$R_1 = \Sigma||F_0|-|F_c||/\Sigma|F_0|$

$wR_2 = \{\Sigma\,[w(|F_0|^2-|F_c|^2)^2/\Sigma[w(F_0^4)]\}^{1/2}$

Table S4. Bond lengths of [H(NO3S)](PF_6).

Bond	Bond length [Å]
P(1)-F(4)	1.567(4)
P(1)-F(7)	1.568(5)
P(1)-F(2)	1.577(5)
P(1)-F(5)	1.580(5)
P(1)-F(6)	1.599(4)
P(1)-F(3)	1.602(4)
N(8)-C(28)	1.485(9)
N(8)-C(17)	1.507(9)
N(8)-C(14)	1.514(10)
N(9)-C(18)	1.454(8)
N(9)-C(19)	1.465(8)
N(9)-C(22)	1.471(8)
N(10)-C(24)	1.463(8)
N(10)-C(27)	1.463(8)
N(10)-C(23)	1.467(9)
S(11A)-C(15)	1.716(10)
S(11A)-C(16A)	1.82(2)
S(11B)-C(16B)	1.82(2)
S(11B)-C(15)	1.957(12)
S(12)-C(20)	1.799(6)
S(12)-C(21)	1.804(7)
S(13)-C(26)	1.785(8)
S(13)-C(25)	1.813(6)
C(14)-C(15)	1.517(12)
C(17)-C(18)	1.517(10)
C(19)-C(20)	1.506(9)
C(22)-C(23)	1.519(9)
C(24)-C(25)	1.525(10)
C(27)-C(28)	1.520(10)

Table S5. Angles of [H(NO3S)](PF_6).

Angle	[°]
F(4)-P(1)-F(7)	179.4(3)
F(4)-P(1)-F(2)	89.5(3)
F(7)-P(1)-F(2)	90.8(4)
F(4)-P(1)-F(5)	90.4(4)
F(7)-P(1)-F(5)	89.3(4)
F(2)-P(1)-F(5)	179.5(3)
F(4)-P(1)-F(6)	90.5(2)
F(7)-P(1)-F(6)	89.9(3)
F(2)-P(1)-F(6)	90.0(2)
F(5)-P(1)-F(6)	90.5(2)
F(4)-P(1)-F(3)	90.1(2)
F(7)-P(1)-F(3)	89.5(3)
F(2)-P(1)-F(3)	90.4(2)
F(5)-P(1)-F(3)	89.2(2)
F(6)-P(1)-F(3)	179.3(2)
C(28)-N(8)-C(17)	111.8(5)
C(28)-N(8)-C(14)	114.4(5)
C(17)-N(8)-C(14)	112.5(6)
C(18)-N(9)-C(19)	111.6(5)
C(18)-N(9)-C(22)	114.2(5)
C(19)-N(9)-C(22)	113.6(5)
C(24)-N(10)-C(27)	114.5(5)
C(24)-N(10)-C(23)	115.1(5)
C(27)-N(10)-C(23)	116.5(5)
C(15)-S(11A)-C(16A)	96.6(7)
C(16B)-S(11B)-C(15)	97.0(9)
C(20)-S(12)-C(21)	100.4(3)
C(26)-S(13)-C(25)	100.5(3)
N(8)-C(14)-C(15)	112.9(6)
C(14)-C(15)-S(11A)	122.6(7)
C(14)-C(15)-S(11B)	102.6(7)
N(8)-C(17)-C(18)	109.8(5)
N(9)-C(18)-C(17)	111.5(5)
N(9)-C(19)-C(20)	113.4(5)
C(19)-C(20)-S(12)	112.4(4)
N(9)-C(22)-C(23)	114.0(5)
N(10)-C(23)-C(22)	113.0(5)
N(10)-C(24)-C(25)	115.6(5)
C(24)-C(25)-S(13)	112.6(5)
N(10)-C(27)-C(28)	108.1(5)
N(8)-C(28)-C(27)	108.5(5)

Table S6. Crystallographic data and refinement details for [Cu(NO3S)][Cu(NO_3)$_4$].

Empirical formula	$C_{15}H_{33}Cu_2N_7O_{12}S_3$	
Formula weight	726.74 g/mol	
Temperature	293(2)	
Radiation and wavelength	Mo-Kα, λ = *0.71073* Å	
Crystal system	Triclinic	
Space group	P-1	
Unit cell dimension	a = 9.423(2) Å<br>b = 11.545(2) Å<br>c = 13.358(3) Å,	α = 96.48(3)°<br>β = 105.41(3)°<br>γ = 94.51(3)°
Volume	1383.1(5) $Å^3$	
Z	2	
Density (calculated)	1.745 Mg/m^3	
Absorption coefficient, μ	1.833 mm^{-1}	
F(000)	748	
Crystal size	0.2 × 0.2 × 0.3 mm	
θ area data collection	1.60 to 25.00°	
Index area	$-12 \leq h \leq 11$, $-15 \leq k \leq 15$, $0 \leq l \leq 17$	
Reflections collected	2515	
Independent reflections	2515 [R(int) = 0.0597]	
Refinement method	Full-matrix least squares on F^2	
Data/restraints/parameters	2437/0/352	
Final R indices [I <2σ(I)]	R_1 = 0.0645, wR_2 = 0.2034	
R indices (all data)	R_1 = 0.1059, wR_2 = 0.2272	
Extinction coefficient	0.002(6)	
Differential signals	0.771 and −1.106 e.$Å^{-3}$	

$R_1 = \Sigma||F_0|-|F_c||/\Sigma|F_0|$

$wR_2 = \{\Sigma[w(|F_0|^2-|F_c|^2)^2/\Sigma[w(F_0^4)]\}^{1/2}$

Table S7. Bond lengths of [Cu(NO3S)][$Cu(NO_3)_4$].

Bond	Bond length [Å]
Cu(1)-N(3)	2.064(12)
Cu(1)-N(2)	2.153(12)
Cu(1)-N(1)	2.186(11)
Cu(1)-S(1)	2.383(4)
Cu(1)-S(2)	2.476(4)
Cu(1)-S(3)	2.727(5)
S(1)-C(8)	1.81(2)
S(1)-C(7)	1.82(2)
S(2)-C(12)	1.78(2)
S(2)-C(13)	1.80(2)
S(3)-C(2)	1.808(14)
S(3)-C(3)	1.81(2)
N(1)-C(1)	1.47(2)
N(1)-C(4)	1.48(2)
N(1)-C(15)	1.53(2)
N(2)-C(9)	1.47(2)
N(2)-C(5)	1.49(2)
N(2)-C(6)	1.51(2)
N(3)-C(14)	1.48(2)
N(3)-C(11)	1.49(2)
N(3)-C(10)	1.50(2)
C(2)-C(1)	1.51(2)
C(4)-C(5)	1.51(2)
C(10)-C(9)	1.52(2)
C(11)-C(12)	1.51(2)
C(7)-C(6)	1.52(2)
C(14)-C(15)	1.50(2)
Cu(2)-O(101)	1.950(11)
Cu(2)-O(104)	1.959(11)
Cu(2)-O(107)	1.968(11)
Cu(2)-O(110)	1.995(11)
Cu(2)-O(110)	1.995(11)
N(101)-O(103)	1.22(2)
N(101)-O(102)	1.25(2)
N(101)-O(101)	1.29(2)
N(102)-O(106)	1.20(2)
N(102)-O(105)	1.22(2)

Table S8. Bond angles of [Cu(NO3S)][Cu(NO_3)$_4$].

Angle	[°]	Angle	[°]
N(3)-Cu(1)-N(2)	84.9(5)	C(14)-N(3)-C(10)	111.3(11)
N(3)-Cu(1)-N(1)	84.1(5)	C(11)-N(3)-C(10)	112.5(11)
N(2)-Cu(1)-N(1)	82.4(4)	C(14)-N(3)-Cu(1)	105.7(9)
N(3)-Cu(1)-S(1)	169.6(3)	C(11)-N(23)-Cu(1)	109.6(9)
N(2)-Cu(1)-S(1)	84.8(3)	C(10)-N(3)-Cu(1)	107.6(9)
N(1)-Cu(1)-S(1)	95.0(4)	C(1)-C(2)-S(3)	114.6(11)
N(3)-Cu(1)-S(2)	85.0(3)	N(1)-C(4)-C(5)	112.7(13)
N(2)-Cu(1)-S(2)	102.1(3)	N(2)-C(5)-C(4)	112.7(13)
N(1)-Cu(1)-S(2)	167.8(4)	N(1)-C(1)-C(2)	114.6(12)
S(1)-Cu(1)-S(2)	96.69(14)	N(3)-C(10)-C(9)	114.1(12)
N(3)-Cu(1)-S(3)	96.8(3)	N(3)-C(11)-C(12)	112.0(12)
N(2)-Cu(1)-S(3)	161.5(3)	C(6)-C(7)-S(1)	111.8(10)
N(1)-Cu(1)-S(3)	79.5(3)	C(11)-C(12)-S(2)	111.6(10)
S(1)-Cu(1)-S(3)	93.2(2)	N(2)-C(6)-C(7)	110.7(12)
S(2)-Cu(1)-S(3)	96.39(14)	N(2)-C(9)-C(10)	109.8(12)
C(8)-S(1)-C(7)	101.3(8)	N(3)-C(14)-C(15)	112.5(13)
C(8)-S(1)-Cu(1)	110.6(6)	C(14)-C(15)-N(1)	111.9(11)
C(7)-S(1)-Cu(1)	98.8(5)	O(101)-Cu(2)-O(104)	90.2(5)
C(12)-S(2)-C(13)	100.8(8)	O(101)-Cu(2)-O(107)	174.9(4)
C(12)-S(2)-Cu(1)	95.6(5)	O(104)-Cu(2)-O(107)	90.8(5)
C(13)-S(2)-Cu(1)	108.3(6)	O(101)-Cu(2)-O(110)	88.6(5)
C(2)-S(3)-C(3)	99.3(8)	O(104)-Cu(2)-O(110)	174.4(5)
C(2)-S(3)-Cu(1)	94.9(5)	O(107)-Cu(2)-O(110)	90.9(5)
C(3)-S(3)-Cu(1)	109.1(7)	O(103)-N(101)-O(102)	122.3(14)
C(1)-N(1)-C(4)	111.5(12)	O(103)-N(101)-O(101)	121.1(14)
C(4)-N(1)-C(15)	110.3(12)	O(102)-N(101)-O(101)	116.5(13)
C(4)-N(1)-C(15)	111.2(12)	N(101)-O(101)-Cu(2)	109.2(9)
C(1)-N(1)-Cu(1)	113.2(9)	O(106)-N(102)-O(105)	127(2)
C(4)-N(1)-Cu(1)	104.1(9)	O(106)-N(102)-O(104)	116.2(14)
C(15)-N(1)-Cu(1)	106.2(8)	O(105)-N(102)-O(104)	116.9(14)
C(9)-N(2)-C(5)	113.4(11)	N(102)-O(104)-Cu(2)	109.2(9)
C(9)-N(2)-C(6)	110.9(11)	O(109)-N(103)-O(108)	123.3(13)
C(5)-N(2)-C(6)	112.1(11)	O(109)-N(103)-O(107)	118.7(13)
C(9)-N(2)-C(6)	112.1(11)	O(108)-N(103)-O(107)	118.0(14)
C(S)-N(2)-Cu(1)	101.8(8)	N(103)-O(107)-Cu(2)	108.5(10)
C(S)-N(2)-Cu(1)	109.7(9)	O(111)-N(104)-O(112)	123.8(14)
C(6)-N(2)-Cu(1)	109.7(9)	O(111)-N(104)-O(110)	118(2)
C(14)-N(3)-C(11)	110.0(11)	O(112)-N(104)-O(110)	118(2)

Table S9. Cathodic peak potential (E_{pc}), anodic peak potential (E_{pa}), difference between E_{pc} and E_{pa} (ΔE_p) and half-wave potential ($E_{1/2}$) for copper complexes of NO3S in aqueous solution at different pH, I = 0.15 mol/L $NaNO_3$, T = 25 °C and v = 0.1 V/s.

pH	E_{pc} [V] *vs.* SCE	E_{pa} [V] *vs.* SCE	ΔE_p [mV] *vs.* SCE	$E_{1/2}$ [V] *vs.* SCE
4.04	−0.070	−0.151	81	−0.111
5.95	−0.081	−0.158	77	−0.119
7.07	−0.084	−0.159	75	−0.121
11.02	−0.113	−0.192	79	−0.152
11.95	−0.160	−0.229	69	−0.195

SCE = saturated calomel electrode

Table S10. Chemical shifts, multiplicities, relative integral, and proton attribution for $[Cu(NO3S)]^+$. Data were taken from **Figure S23**.

Chemical shift [ppm]	Multiplicity	Relative integral	Proton Attribution
2.22	s	9	SCH_3
2.65	m	6	NCH_2 macrocycles
2.80	m	6	NCH_2 macrocycles
2.87	t	6	SCH_2
2.94	t	6	NCH_2 arms

s = singlet; m = multiplet; t = triplet

Supplementary References

(1) Tosato, M.; Dalla Tiezza, M.; May, N. V.; Isse, A. A.; Nardella, S.; Orian, L.; Verona, M.; Vaccarin, C.; Alker, A.; Mäcke, H.; Pastore, P.; Di Marco, V. Copper Coordination Chemistry of Sulfur Pendant Cyclen Derivatives: An Attempt to Hinder the Reductive-Induced Demetallation in $^{64/67}Cu$ Radiopharmaceuticals. *Inorg. Chem.* **2021**, *60* (15), 11530-11547.

(2) Tosato, M.; Pelosato, M.; Franchi, S.; Isse, A. A.; May, N. V.; Zanoni, G.; Mancin, F.; Pastore, P.; Badocco, D.; Asti, M.; Di Marco, V. When Ring Makes the Difference: Coordination Properties of Cu^{2+}/Cu^+ Complexes with Sulphur-Pendant Polyazamacrocycles for Radiopharmaceutical Applications. *New J. Chem.* **2022**, *46*, 10012-10025.
